# TiO_2_ doped chitosan/hydroxyapatite/halloysite nanotube membranes with enhanced mechanical properties and osteoblast-like cell response for application in bone tissue engineering†

**DOI:** 10.1039/c9ra08366a

**Published:** 2019-12-02

**Authors:** Sarim Khan, Viney Kumar, Partha Roy, Patit Paban Kundu

**Affiliations:** Department of Chemical Engineering, Indian Institute of Technology Roorkee 247667 India ppkfch@iitr.ac.in sarimk@mit.edu; Department of Biotechnology, Indian Institute of Technology Roorkee 247667 India; Institute for Medical Engineering and Science, Massachusetts Institute of Technology Cambridge USA

## Abstract

The current therapeutic strategies for healing bone defects commonly suffer from the occurrence of bacterial contamination on the graft, resulting in nonunion in the segmental bone defects and the requirement for secondary surgery to remove or sterilize the primary graft. A membrane with enhanced anti-bacterial efficacy, mechanical strength and osteoconductivity would represent an improvement in the therapeutic strategy for guided bone regeneration. The present study aims to optimize the content of halloysite nanotubes (HNTs) and TiO_2_ in the polymer matrix of chitosan (CTS) with a constant amount of nano-hydroxyapatite (5%) with the objective of mimicking the mechanical and biological microenvironment of the natural bone extracellular matrix with enhanced anti-bacterial efficacy. HNTs are a low-cost alternative to MWNCTs for enhancing the mechanical properties and anti-bacterial efficacy of the composite. From the first stage of the study, it was concluded that the membranes possessed enhanced mechanical properties and optimum biological properties at 7.5% (w/w) loading of HNTs in the composite. In the second stage of this investigation, we studied the effect of the addition of TiO_2_ nanoparticles (NPs) and TiO_2_ nanotubes (NTs) in small amounts to the CTS/n-HAP/HNT nanocomposite at 7.5% HNT loading, with an aim to augment the anti-bacterial efficacy and osteoconductivity of this mechanically strong membrane. The study revealed a significant enhancement in the anti-bacterial efficacy, osteoblast-like MG-63 cell proliferation and ALP expression with the addition of TiO_2_ NTs. The CHH-TiT membrane successfully inhibited the *S. aureus* and *E. coli* growth within 16 hours and simultaneously assisted the enhanced proliferation of osteoblast-like cells on its surface. The study supports the potential exploitation of CHH-TiT (7.5% HNT & 0.2% TiO_2_ NT) membranes as a template for guided bone tissue regeneration.

## Abstract 2

This two-stage study aims to optimize the amount of halloysite nanotubes and TiO_2_ in a chitosan/nano-hydroxyapatite composite to tailor the mechanical and biological properties for application in bone tissue engineering.

## Graphical abstract

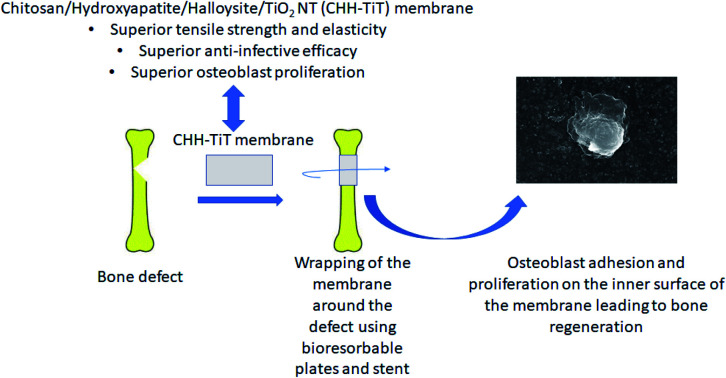

## 1. Introduction

Bone grafts are typically utilized in an extensive number of clinical settings to supplement bone healing and regeneration. There are two types of traditional bone grafting methods, namely autografts and allografts.^1^ Autografts are histocompatible and non-immunogenic, but they require secondary surgery to harvest the graft from the patient's own iliac crest.^2,3^ Thus, they are very expensive and are associated with complications such as donor site injury and morbidity, inability to seal gaps due to a lack of bone graft harvested from the iliac crest and the possibility of infections.^4,5^ Allografts are the second most common grafting technique employed; although they are histocompatible, they suffer from the risks of transmission of infections and immunoreactions.^1^ Allografts are devitalized before grafting them, so they have diminished osteoconductive properties.^6^ The field of bone tissue engineering focuses on alternative treatment methods by grafting a biocompatible and osteoinductive template that closely mimics the natural bone extracellular matrix and assists in bone tissue regeneration.^1^ Grafting a polymer–nanoceramic composite material does away with the complications associated with the traditional clinical grafting methods such as donor site morbidity, the limited supply of grafting material, high cost and immunogenic rejection. An ideal bone tissue nanocomposite should be multi-functional in nature, it should possess properties such as suitable mechanical strength, biocompatibility, antibacterial efficacy, biodegradability, hemocompatibility, and surface functionality to favor cell proliferation.^7,8^ The most commonly used constituents in polymer–ceramic nanocomposites are chitosan (CTS) and nano-hydroxyapatite (n-HAP, Ca_10_(PO_4_)_6_(OH)_2_), due to their excellent biocompatibility with the human body.^9^ Chitosan has several important properties such as antibacterial efficacy, biocompatibility, biodegradability, and cytocompatibility.^10^ Chitosan is non-toxic and linear in nature and is made up of randomly dispersed β-(1→4)-linked d-glucosamine and *N*-acetyl-d-glucosamine. It is synthesized by alkali deacetylation of the chitin shells of crustaceans.^11^ It has been reported that *in vivo* chitosan biodegrades to non-toxic products by the enzymatic action of lysozyme or chitosanase.^12^ The degradation kinetics is governed inversely by the crystallinity and degree of acetylation of the chitosan polymer.^12^ But chitosan has limited osteoconductivity, therefore there is a need to identify nanofillers that can increase the osteoconductivity. Nano-hydroxyapatite (n-HAP) is an obvious choice as it provides ample calcification sites thereby increasing the osteoconductivity of the nanocomposite.^13^ n-HAP has been reported to increase the attachment and proliferation of neonatal rat osteoblast cells.^14^ But the addition of HAP decreases the tensile strength of the nanocomposite as n-HAP is intrinsically brittle in nature. Halloysite nanotubes are natural and inorganic multi-walled nano-dimensional tubes and are mined from natural deposits. Their molecular formula is Al_2_Si_2_O_5_(OH)_4_·*n*H_2_O.^15^ They possess a high aspect ratio which helps in reinforcing a polymer by optimizing the load transfer. In addition, they are reported to be thermally stable and biocompatible.^15^ HNTs are non-toxic, abundant in nature and cheap to procure, so they are a viable alternative to MWCNTs for use in bone tissue engineering.^16^ Alongside n-HAP, HNTs can also be incorporated into the CTS polymer matrix to improve its mechanical properties to mimic the natural bone extracellular matrix. TiO_2_ nanoparticles (NP) are classified as a bio-ceramic which possess appreciable mechanical strength and toughness and have been in considerable attention because of their capability to enhance osteoblast adhesion and to induce cell growth by establishing a chemical bond with the living bone tissue.^17^ TiO_2_ nanotubes (NT) are one dimensional (1-D) nanotubes that possess a very large surface area and it has been reported that they are more osteoconductive than their nanoparticle counterpart.^18^ The effect of the separate additions of TiO_2_ NP and TiO_2_ NT into a chitosan matrix containing n-HAP is yet to be reported in the literature, moreover, these nanofillers can be used to augment the mechanical and biological properties of the chitosan composites. The current clinical grafting methods suffer from post-operative infections and the unwarranted adhesion between the healing bone and the adjacent soft tissues. A membrane with enhanced anti-bacterial efficacy would fight-off any post-operative bacterial infections on their own without any secondary interventions or surgeries, thus helping reduce the cost and the healing time. A mechanically strong membrane would provide a barrier for maintaining the original shape of the bone and avert any postoperative attachment between the bone and the surrounding soft tissues.^19,20^ These two associated complications with the current standards have inspired this study. The ultimate tensile strength of the human trabecular bone is around 50 MPa.^21^ Chitosan polymer matrix is reported to have poor mechanical strength and elasticity, which restricts its use in bone tissue regeneration.^22^ One of the major aims for the nanofiller addition in this study is to propel the mechanical strength of the chitosan matrix well above the strength and elasticity of natural bone so that adequate mechanical support can be provided even during the biodegradation phase of the polymer matrix. Liu *et al.*, have studied the chitosan–halloysite interactions,^26,29^ but their studies have not investigated the application of the membranes in bone tissue regeneration. Moreover, the addition of n-HAP is crucial for a bone tissue template. From their studies, it is evident that the fibroblast proliferation at the synthesized membranes of CTS/HNT is lower than that for the control. In this study, we sought to engineer and augment the osteoblast-like cell response of CTS/n-HAP/HNT with the addition of TiO_2_ after establishing the optimised amount of HNT in the composite.

In this study, we have two different hypotheses. First, we set out to fabricate a nanocomposite series of CTS/n-HAP/HNT (CHH I-III), which is yet to be reported in the literature. Here, our hypothesis is that mechanical and biological properties of the membrane would depend on the HNT loading in the membrane and these properties would be more favorable in comparison to CTS/n-HAP nanocomposite, so the loading of HNTs has been varied (5%, 7.5%, and 10%; w/w) to study the effect of HNT concentration on the mechanical and biological characteristics and the relative agglomeration of HNTs with increasing concentration in the membranes. The content of n-HAP was kept constant at 5% for all the membranes.^7,23^ The primary aim was to discover the membrane with the most favorable mechanical properties and optimum biological properties amongst CHH I–III. The HNT loading in that membrane would be considered as the optimized HNT loading in CTS/n-HAP (5%; w/w)/HNT nanocomposites for mimicking natural bone extracellular matrix. Subsequently, our second hypothesis is that the separate additions of TiO_2_ NP (0.2%) and TiO_2_ NT (0.2%) particles to the CTS/n-HAP/HNT membrane with optimized HNT loading would enhance the biological properties of the membrane. Then we compared the changes in mechanical and biological properties upon TiO_2_ nanoparticle addition (CHH-TiP membrane) and TiO_2_ nanotube (CHH-TiT membrane) addition to the nanocomposite. The major outline of this study is to engineer a chitosan-based polymer nano-construct with enhanced mechanical strength, elasticity, anti-bacterial efficacy, cell proliferation and attachment using nanofillers and to ensure that these membranes can be used for bone regeneration applications which would not be possible with a bare CTS polymer membrane due to several shortcomings as highlighted above. The fabricated nanocomposites have been characterized by Fourier transform infrared spectroscopy (FTIR) and small-angle X-ray diffraction (XRD) spectroscopy. The surface morphology, topographical features, and agglomeration of HNTs have been studied using field emission scanning electron microscopy (FESEM), transmission electron microscopy (TEM) and atomic force microscopy (AFM). The mechanical, thermal, water absorption, pH, hemocompatibility antimicrobial, cell proliferation, attachment and differentiation studies have been carried out in order to establish the potential of the fabricated membranes for further *in vivo* investigations to be employed as a bone defect healing template.

## 2. Methodology

### 2.1 Synthesis of TiO_2_ nanotubes, nano-HAP-TiO_2_ NP and nano-HAP-TiO_2_ NT

The TiO_2_ nanotubes were synthesized from TiO_2_ nanoparticles (Sigma Aldrich, 99%) by hydrothermal treatment using a standard procedure.^24^ To synthesize nano-HAP, we followed the wet precipitation method.^22^ Since TiO_2_ is not readily soluble in aqueous solutions, separate fused mixtures with n-HAP were formed for both TiO_2_ NP and NT. To synthesize nano-HAP-TiO_2_ NP and nano-HAP-TiO_2_ NT, 4% (w/w) of TiO_2_ NP and 4% (w/w) of TiO_2_ NT were added separately to 96% (w/w) of nano-HAP aqueous solution and the mixture was stirred at 800 rpm for 2 h. The resulting suspension was then filtered and dried in a hot air oven at 100 °C for 2 h.

### 2.2 Synthesis of CTS/n-HAP/HNT and CTS/n-HAP-TiO_2_/HNT nanocomposites

To synthesize the CTS/n-HAP/HNT nanocomposite membranes CH I-III (Table 1), we followed the solution casting method.^46^ Briefly, we took varying percentages of CTS (85–90%) (w/w), HNT (5–10%) (w/w), nano-HAP (5%) (w/w) for the membranes. For CHH-TiP and CHH-TiT (Table 1), we added nano-HAP-TiO_2_ NP (5%) and nano-HAP-TiO_2_ NT (5%) respectively instead of n-HAP. The total amount of TiO_2_ NP in CHH-TiP and TiO_2_ NT in CHH-TiT was 0.2%. Briefly, the required amount of chitosan (HiMedia Private Ltd, degree of deacetylation ≥75%; *M*_w_ ∼ 150–200 kDa) was added to aqueous solution of 2.5% (v/v) glacial acetic acid (HiMedia Private Ltd) and subsequently stirred for 2 hours at 800 rpm at room temperature. HNT (Sigma Aldrich), nano-HAP, nano-HAP-TiO_2_ NP or nano-HAP-TiO_2_ NT were then added in the required amounts to the solution and stirred continuously for another 8 h at 800 rpm. The excess acetic acid in the formulation was partially neutralized using 0.1 N NaOH (HiMedia Private Ltd) solution. Subsequently, the solution was ultra-sonicated at 200 watts for 1 h to get a homogenous blend with proper intercalation. The formulation was then poured into a Petri-dish and the solvent was allowed to evaporate. After complete evaporation, the membrane was dried at 60 °C for another 24 h. A control sample (CH) containing only CTS and nano-HAP (5%), was synthesized using the above-described procedure.

**Table 1:** Classification of CHH I–III, CHH-TiP, CHH-TiT and CH

	CTS (%)	HNT (%)	n-HAP (%)	HAP-TiO_2_ NP (%)	HAP-TiO_2_ NT (%)
CHH I	90	5	5	0	0
CHH II	87.5	7.5	5	0	0
CHH III	85	10	5	0	0
CHH-TiP	87.5	7.5	0	5	0
CHH-TiT	87.5	7.5	0	0	5
CH	95	0	5	0	0

### 2.3 Experiments and characterization

X-ray diffraction (XRD) study of the CHH I–III, CHH-TiP, CHH-TiT, and CH were recorded using Glancing Angle XRD (Bruker Model- D8-Advance) and patterns of HNT, HAP, n-HAP-TiO_2_ NP and n-HAP-TiO_2_ NT were recorded using Powder X-ray Diffractometer (Bruker Model- D8-Advance). Cu K_α_ radiations were employed in the scanning range of 5° to 70° with the generator operating at 40 kV and 30 mA. The step size was 0.02° with a constant exposure time of 5 s per step. All the samples were studied in triplicates and a representative figure has been presented.

Fourier transform infrared (FTIR) spectra were recorded for thin dry membranes and powder using FT-IR spectrometer (PerkinElmer) in ATR mode. Dry membranes of CHH I–III, CHH-TiP, CHH-TiT, CH and powders of HNT, HAP, HAP- TiP, and HAP- TiT were scanned in the wavenumber range of 4000–400 cm^−1^ at a resolution of 4 cm^−1^. The spectra for each sample were recorded in triplicates. No significant spectral variations for the samples were encountered, so, a representative FT-IR spectrum has been presented for each of them.

Surface morphology analysis of CHH I–III, CHH-TiP, CHH-TiT membranes and size analysis of nano-HAP, TiO_2_ nanoparticle were investigated using Carl-Zeiss Field-Emission Scanning Electron Microscope (ZEISS Gemini SEM 300). The bulk microstructure and the agglomeration of the HNTs in CHH I–III were investigated using a transmission electron microscope (FEI Tecnai G2 20 S-Twin). The solutions of the polymer formulations were diluted with ethanol and then drop casted (5 μL) onto the copper grid. They were subsequently imaged after the solvent evaporated. The structure and size of HNTs and TiO_2_ nanotubes were also investigated using TEM. In addition, to record the nano-topographic features of the dry nanocomposite membranes (CHH I–III), we used a scanning probe microscope (NT-MDT-INTEGRA) with AFM module.

These analyses were carried out in triplicates, but no significant variation was encountered. So, the representative figures for these samples have been provided here.

Mechanical properties such as tensile strength, Young's modulus, stiffness and the elongation at break of CHH I–III, CHH-TiP, CHH-TiT, and CH were recorded using the Small Scale Mechanical Tensile Tester for Biomaterials (Bose ElectroForce 3200 Series III), at a crosshead speed of 5 mm min^−1^. The sample membranes had a gauge length of 20 mm, a width of 5 mm and a thickness of 0.06 mm.

Thermogravimetric analysis (TGA) for CHH II, CHH-TiP, CHH-TiT and CH membranes was carried out in a thermo-gravimetric and differential thermal analyzer (TG-DTA, SII 6300 EXSTAR). 10 mg of sample was placed on a platinum and alumina pan and was then gradually heated from 35 °C to 620 °C at a controlled heating rate of 20 °C min^−1^. The heating was carried out in a nitrogen atmosphere to provide an inert atmosphere without oxygen. The flow rate of the carrier gas (N_2_) was fixed at 200 mL min^−1^ (STP). Finally, based on the residual weight of the nanocomposite with temperature, we carried out the thermogravimetric analysis (TGA) of the nanocomposite samples.

Rheological measurements of the nanocomposite membranes such as storage modulus (*E*′) and loss modulus (*E*′′) were evaluated using a Dynamic Mechanical Analyzer (TA Q800) in the tensile mode. The dry sample membranes had a gauge length of 30 mm, a width of 5 mm and a thickness of around 0.06 mm. A frequency sweep was employed to record changes in the storage modulus (*E*′) and loss modulus (*E*′′) in the frequency range of 0.1–5 Hz at room temperature (25 °C) under a constant strain of 0.1 Hz.

Water absorption studies of all the fabricated membranes were carried out to measure the ease of cell infiltration in the membranes. The experiment procedure has been described in SI 1.1 in ESI.† Finally, the percentage of water absorption (*E*_a_) was calculated using the following equation:*E*_a_ = (*W*_t_ − *W*_0_)/*W*_0_ × 100

pH studies of all the membranes were carried out, 8 mg of each membrane sample was mixed with 30 mL of freshly prepared physiological saline solution (0.9% NaCl). The prepared solutions were stirred continuously at 37 °C on a magnetic stirrer. The pH values were recorded after 1, 2, 4, 7, and 14 days using a pH meter.

Hemolytic assay of CHH I–III, CHH-TiP, CHH-TiT and CH membranes were carried out with fresh human blood to establish their compatibility with human erythrocytes. The experiments were approved by the Institute Human Ethics Committee of IIT Roorkee and were carried out as per the set guidelines. The informed consent was obtained from the human participants of this study. The standard procedure for the hemolytic assay was followed (ASTM F756-00) (SI 1.2 in ESI†). Finally, the percentage of hemolysis for each sample is calculated using the formula:Hemolysis (%) = (OD(sample) − OD(negative control)) × 100/(OD(positive control) − OD(negative control))

Antibacterial efficacy of CHH I–III, CHH-TiP, CHH-TiT, and CH were investigated against the bacterial strain of *Escherichia coli* XL1B (Gram negative) and *Staphylococcus aureus* (Gram-positive), using the liquid culture method^25^ (SI 1.3 in ESI†). Images supplied are representatives of three independent experiments.

Ion release study was carried out to measure the release of Al and Si ions from the membranes, 8 mg of each of the CHHI-III and CH film samples were immersed in 30 mL of fresh phosphate-buffered saline (PBS) solution at 37 °C for 7 days. Then the solution was collected for inductively coupled plasma (ICP) emission spectral analysis ((PerkinElmer) ELAN DRC-e) to detect the concentration of silicon (Si) and aluminum (Al) ions released from the HNTs in CHH I–III.

*In vitro* cell proliferation study (MTT assay) was carried out to investigate the cytocompatibility of osteoblast-like MG-63 cells with the composites CHH I–III, CHH-TiP, CHH-TiT, and CH. The MG-63 cells have the potential to retain a differentiated phenotype in culturing conditions and they proliferate faster compared to normal osteoblast cells. The cells were harvested by a 3 min trypsinization with 0.25% trypsin–EDTA solution (Sigma Aldrich; T4049) followed by centrifugation. MTT assay was carried out following a standard procedure (SI 1.4 in ESI†) on the 1^st^ and the 3^rd^ day.

Cell attachment and morphological study with the osteoblast-like MG-63 cells were carried out, the membranes with the seeded cells were rinsed thrice with PBS and then fixed using 4% paraformaldehyde at 4 °C for 30 minutes after 24 h and 72 h of initial seeding. Then the membranes were sequentially immersed in 25%, 50%, 75% and 100% ethanol for dehydration. The membranes were subsequently imaged using Carl-Zeiss Field-Emission Scanning Electron Microscope (ZEISS Gemini SEM 300). Images provided are representatives of three independent experiments.

Alkaline phosphatase (ALP) activity test was carried out with the osteoblast-like MG-63 cells to measure the extent of ALP expression after 3 and 7 days initial seeding on the membranes. The UV-sterilized membrane samples were cultured with 5 × 10^3^ MG-63 cells each in a 48 well plate. The test was performed following a standard procedure (SI 1.5 in ESI†). The ALP activity has been normalized for each well by dividing it by the protein content in the well to account for different cell proliferation.

All the quantitative data yielding experiments (mechanical properties, rheological measurements, water absorption, pH study, hemolytic assay, antibacterial efficacy, MTT assay, and ALP activity test) were carried out in triplicates and their reported values are expressed as the means of triplicate counts ± standard deviations (SD). Except for TGA, which was only carried out once. Statistical analysis was conducted using the ANOVA test (Dunnett's T3 multiple comparisons test).

## 3. Results and discussion

### 3.1 Optimization of the HNT loading in CTS/n-HAP/HNT (CHH I–III) nanocomposite membranes

The XRD patterns of CHH I–III are shown in SI 3.1 in ESI,† the presence of CTS is characterized by the peaks at 2*θ* = 9.47° and 20.08°. The broad peak at 20.08° demonstrates the amorphous nature of CTS. The peak intensity increases with an increasing amount of CTS in the nanocomposite. These peaks at 9.47° and 20.08° correspond to planes (020) and (022). The presence of HNT is marked by peaks at 2*θ* = 12°, 20° and 25°,^26^ corresponding to (001), (02, 11) and (002) planes, respectively. The intensity of the peak at 2*θ* = 20° increases with increasing HNT content. The presence of n-HAP is confirmed from strong peaks at 2*θ* = 31.76°, 32.97°and additional peaks are also observed at 25.74° and 49.41° (SI 3.3 in ESI†), which are in good accordance with the standard ICDD card # 09-0432.

**Fig. 1:**
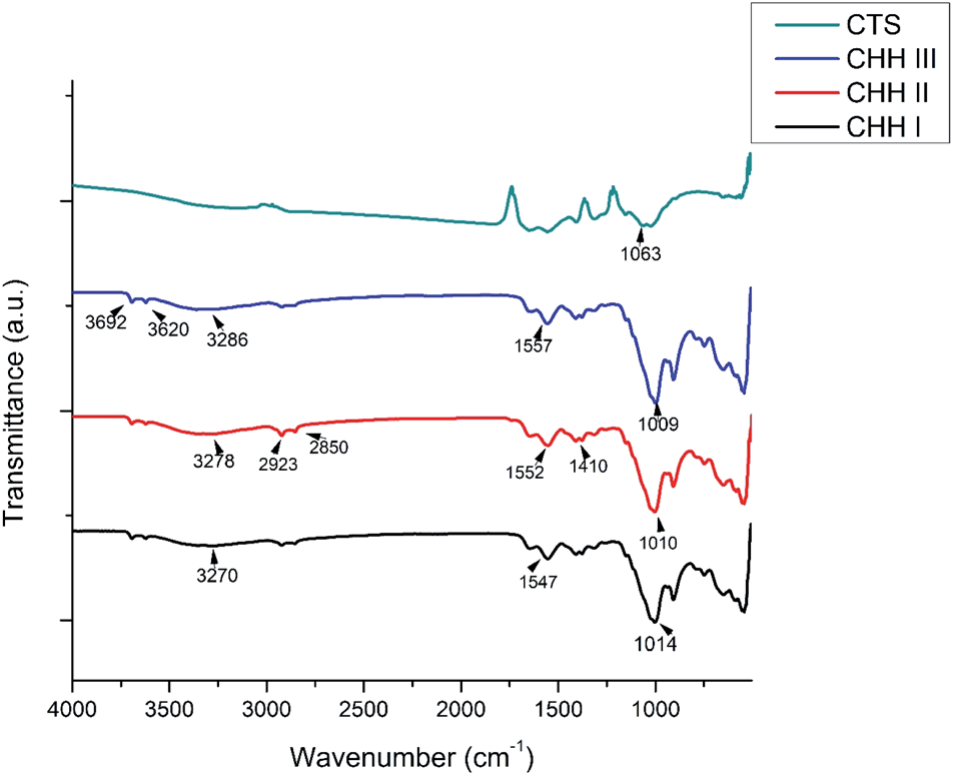
FTIR patterns of CHH I–III and chitosan (CTS).

From the FTIR spectra of CHH I–III (Fig. 1), the presence of chitosan is confirmed from the broad peaks at 3270 cm^−1^, 3278 cm^−1^, and 3286 cm^−1^, which are attributed to the overlapped NH band and OH band vibration.^27^ This shift in the frequencies in CHH I–III suggests the presence of electrostatic interaction and hydrogen bonding between the HNTs and chitosan.^28,29^ A similar change in the peaks centered at 1552 cm^−1^ and 1410 cm^−1^ was observed; these peaks can be attributed to the deformation vibration of the hydroxyl group and protonated amine group in chitosan.^27^ The presence of HNTs can be established by the presence of two peaks centered at 3692 cm^−1^ and 3620 cm^−1^, due to the Al_2_-OH stretching bands of HNTs.^26^ Also, the broad peaks centered at 1014, 1010 and 1009 cm^−1^ for CHH I–III respectively, were present due to the overlap of phosphate stretching vibration of n-HAP^27^ and the glycosidic linkage present in chitosan.

**Fig. 2:**
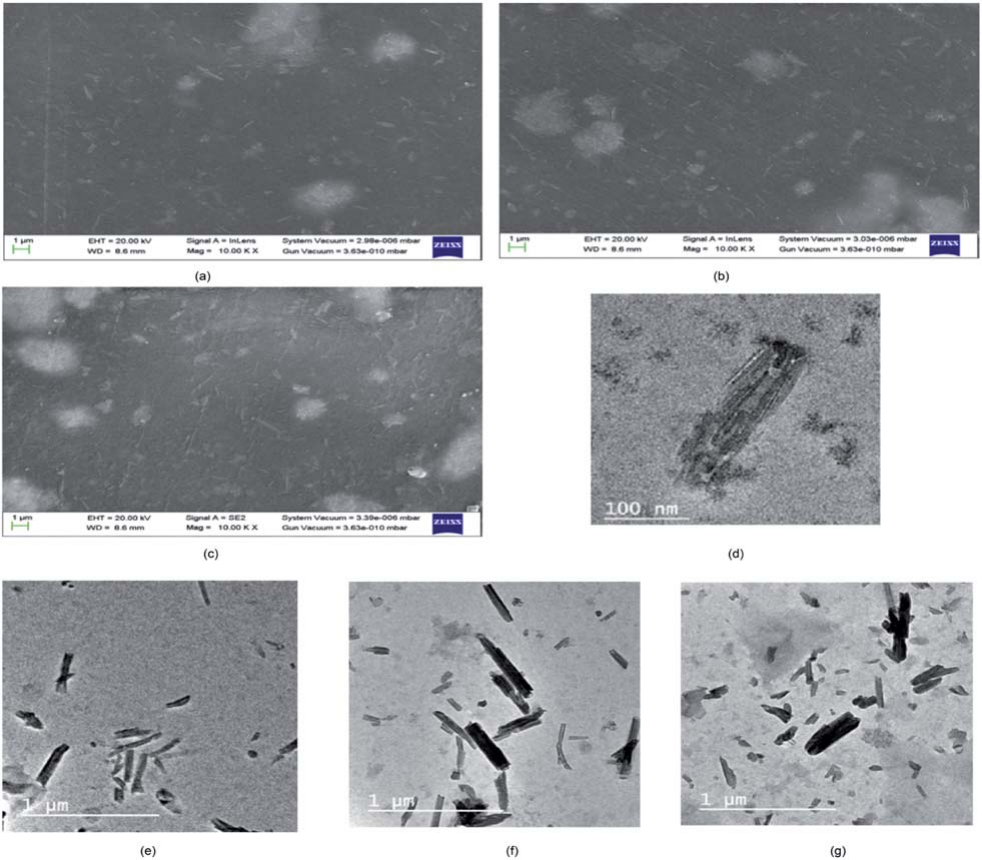
FESEM images of CHH I (a), CHH II (b), CHH III (c) and TEM images of TiO_2_ nanotube (d), CHH I (e), CHH II (f) and CHH III (g).

**Fig. 3:**
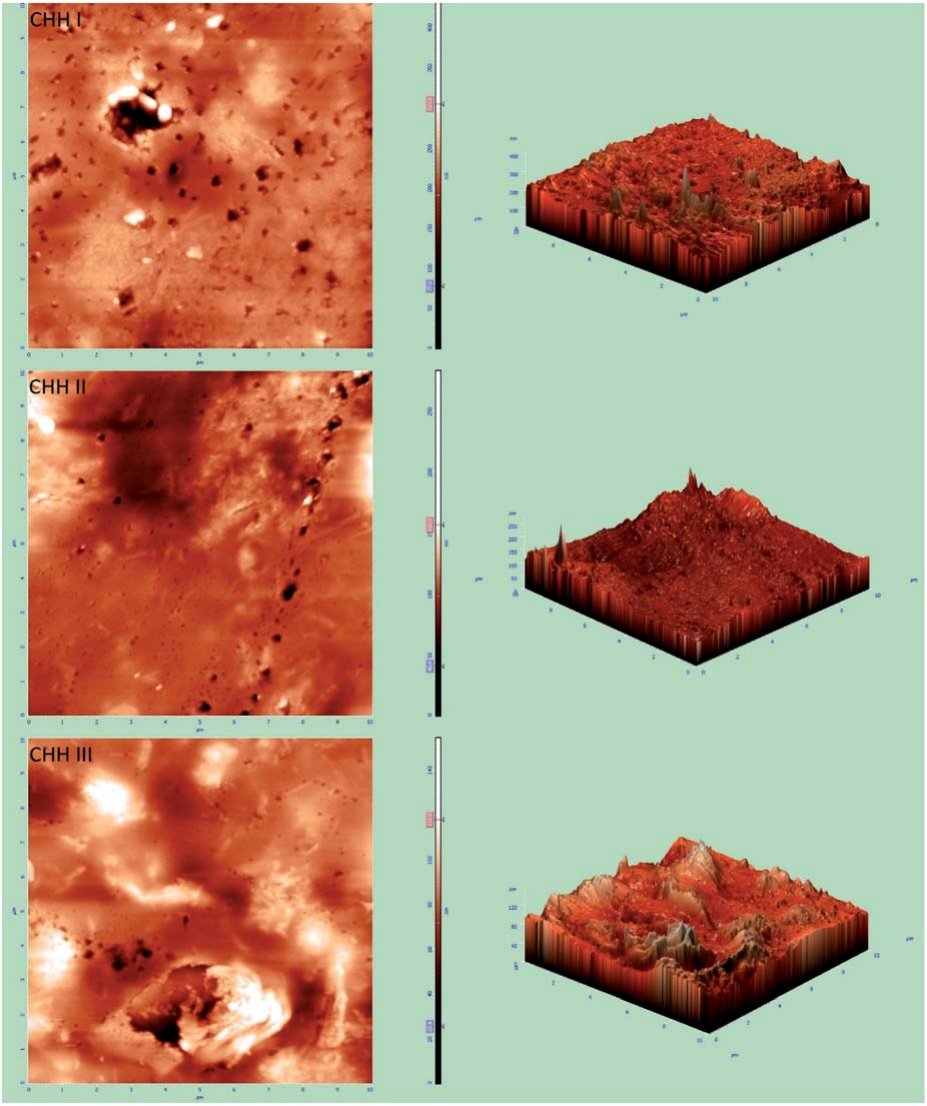
AFM images of the surfaces of CHH I–III (top to bottom).

From the FESEM image (SI 3.5 in ESI†), it can be confirmed that the nano-HAP particles are spherical in shape and uniform in size, with the particle diameter varying in the narrow range of 18–25 nm. The HNTs are tube-shaped (SI 3.6 in ESI†) with varying lengths in the range of 200–600 nm and an average diameter of 70 nm. From the FESEM images of CHH I–III (Fig. 2(a)–(c)), it is observed that as the percentage loading of HNT increases from CHH I (5%) to CHH III (10%), the agglomerated states of these tubes increase in number and size. CHH I (5% HNTs) shows the most homogenous dispersion of HNTs, while CHH II (7.5% HNTs) has both well-dispersed tubes as well as smaller aggregated states of the dimensions 1.5 μm × 1.5 μm. CHH III (10% HNTs) has relatively larger agglomerate centers which can act as fracture points or stress concentration points during mechanical loading. The major reason for the agglomeration of these tubes is attributed to the re-aggregation during the solvent evaporation in the solvent casting method employed. The uniform distribution of HNTs in CHH I and CHH II can also be confirmed from their TEM images (Fig. 2(e)–(g)). On the other hand, the presence of agglomerates of HNTs in a small number and size in CHH II and a large number and size in CHH III is confirmed from their respective TEM images. The regions surrounding the HNTs in highly magnified TEM images of CHH II are blurred at several interface points in the TEM image (SI 3.9 in ESI†); this can be due to the wrapping of chitosan around the HNTs, thus providing evidence for the presence of interaction between the HNTs and the chitosan polymer matrix. Similar interactions have also been reported in a similar study of the CTS matrix and HNT.^29^ The EDX (SI 3.10 in ESI†) analysis confirms the presence of n-HAP and HNTs in CHH II. Upon examining the nano-topography results of the CTS/n-HAP/HNT nanocomposites (Fig. 3), it is seen that there is a non-uniform distribution of nanopores in CHH I–III membranes. Such nanopores were absent in a CTS-HNT nanocomposite synthesized by Liu *et al.*, 2012.^26^ The pores present might assist in the transfer of cell nutrition and oxygenation through the process of diffusion as the membrane thickness is 60 μm, which is well below the diffusion limit of oxygen (200 μm).^30^ The surface roughness of these nanocomposites also increases with the increasing amount of HNTs in the nanocomposites which are present at the surface of the membrane. The average surface roughness values for CHH I–III are 4.58 nm, 9.60 nm, and 17.20 nm respectively. The increasing surface roughness is advantageous for cell attachment and proliferation. The positively charged chitosan in the mildly acidic solution interacts electrostatically with the HNTs in the polymer matrix, so no surface modification of HNTs was required in order to disperse them in the polymer matrix.^28^

The trabecular bone is reported to have a tensile strength of 50 MPa,^21^ so the tensile strength of the membrane swathed around the healing bone should be of similar magnitude. The mechanical properties such as ultimate tensile strength (UTS), stiffness, Young's modulus, and elongation at break of CHH I–III and CH were evaluated in tensile mode and are listed in Table 2. The ultimate tensile strength is maximum for CHH II, amongst CHH I–III which is attributed to the uniform dispersion of the HNTs in CHH II. The UTS of CHH II (7.5% HNT) is significantly greater than CHH I (5% HNT) due to the higher loading of a high aspect nano-filler (HNTs) which optimizes the load transfer from the polymer matrix to the tube. But due to the excessive agglomeration of HNTs in CHH III (10% HNT), there is a large number of stress concentration points which act as fracture points during stress application, but interestingly the mechanical strength of CHH III is greater than the control sample (CH) which doesn't contain any HNTs. The elongation at break (%) was lesser for CHH I–III in comparison to CH, as the strength and toughness have a trade-off relationship. The Young's modulus and stiffness followed a similar trend as the ultimate tensile strength for the CHH I–III nanocomposites. To assess the gain in the thermal stability of the membrane due to the addition of HNTs, the results of TGA have been analyzed. From SI 3.11 and SI 2 in ESI,† it is observed that the primary degradation occurs between the temperature range of 100–200 °C due to the loss of solvent molecules, while the secondary degradation occurs in the temperature range of 250–300 °C. Also, the thermal degradation of CHH II is protracted (SI 3.11 in ESI†) in comparison to CH (control samples), due to the addition of 7.5% (w/w) HNTs in CHH II. The additional thermal resistance due to the presence of HNTs results from the tubular structure of HNTs and the presence of iron.^31^

**Table 2:** Mechanical properties of CHH I–III, CHH-TiP, CHH-TiT, and CH^a^

	Ultimate tensile strength (MPa)	Elongation at break (%)	Young's modulus (MPa)	Stiffness (kN m^−1^)
CHH I	44.29 ± 1.82	35.45 ± 1.36*	641.48 ± 20.17	90.50 ± 3.09
CHH II	58.91 ± 2.13*	38.46 ± 1.11*	836.25 ± 31.89*	115.97 ± 4.17
CHH III	40.87 ± 2.01	29.32 ± 3.12**	502.15 ± 28.65	80.64 ± 10.21
CHH-TiP	64.36 ± 2.74*	42.88 ± 2.07	932.26 ± 39.76**	132.23 ± 7.98*
CHH-TiT	67.14 ± 2.51*	37.72 ± 1.94*	967.49 ± 37.70**	140.14 ± 5.61*
CH	36.88 ± 1.44	52.29 ± 3.55	454.63 ± 20.43	90.70 ± 14.43

a Data here is represented as means of triplicate values ± SD, wherein * indicates *p* < 0.05 and ** indicates *p* < 0.01 when compared with CH.

**Fig. 4:**
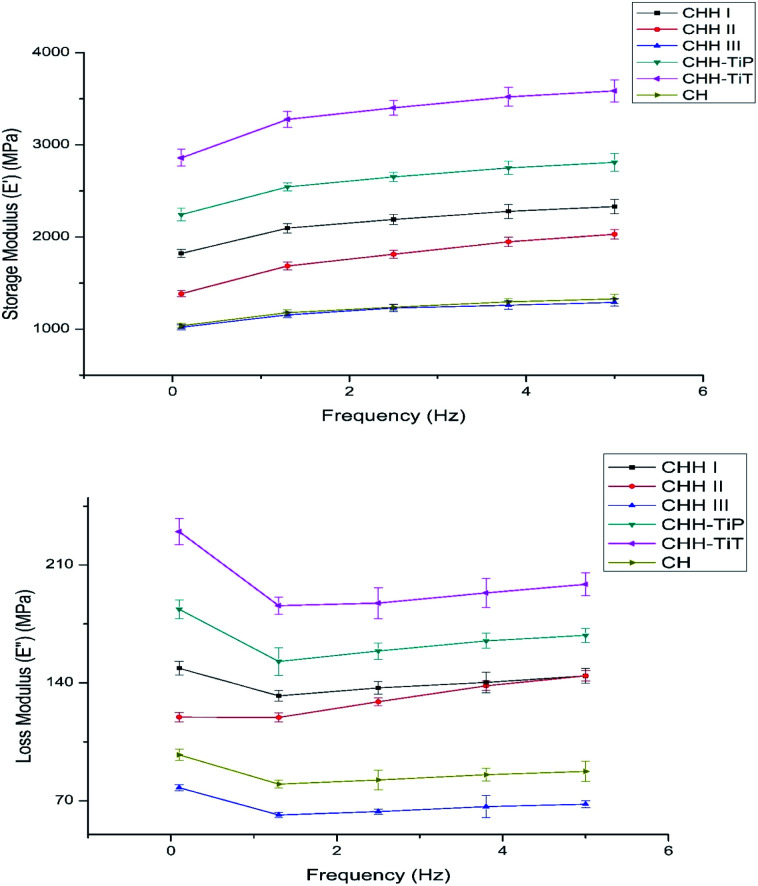
Variation of storage (*E*′) and loss (*E*′′) modulus with frequency for CHH I–III, CHH-TiP, CHH-TiT, and CH. Data here is represented as means of triplicate values ± SD.

The membranes used for bone regeneration are expected to experience a stimulus when implanted *in vivo* due to daily activities. Thus, it is crucial to assess the viscoelastic nature of the membranes to ensure that the membranes return to their original state after experiencing such a stimulus. To evaluate the effect of HNT loading on the network structure of the CTS/n-HAP/HNT nanocomposite, an analysis of the rheological measurements (Fig. 4) was carried out. The storage modulus (*E*′) increased with increasing frequency for CHH I–III membranes, suggesting the viscoelastic nature of CHH I–III membranes. The tube–tube interaction for HNTs is minimal at low loading (5%), but it dominates at relatively higher loading (>10%). Significantly higher *E*′ for CHH I and CHH II over that of CHH III are attributed to the better dispersion and better interfacial interactions of HNTs with CTS polymer matrix, resulting in enhanced elasticity. CHH III has a lower *E*′ than the control sample (CH) due to the presence of large agglomerate states. The tube–tube interaction is significant at a higher loading (10% HNTs), resulting in the agglomeration of HNTs and a decreasing elasticity of the system thereafter. A larger value of storage modulus for the nanocomposites at a higher frequency in comparison to that at lower frequencies indicates that the polymer nanocomposite is taking lesser time for re-orientation at higher frequencies after providing a frequency driven deformation stimulus to the membranes.

**Fig. 5:**
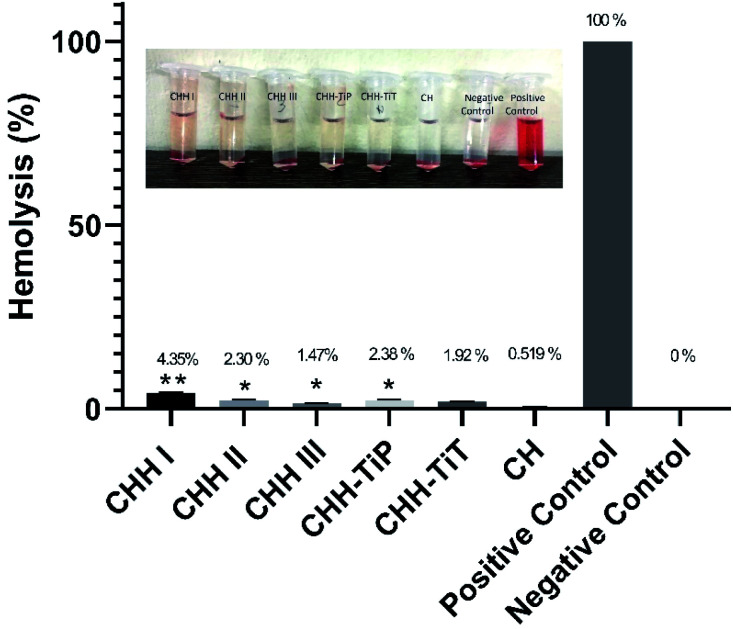
Hemolysis study of CHH I–III, CHH-TiP, CHH-TiT and CH. Data here is represented as means of triplicate values ± SD, wherein * indicates *p* < 0.05 and ** indicates *p* < 0.01 when compared with CH.

**Fig. 6:**
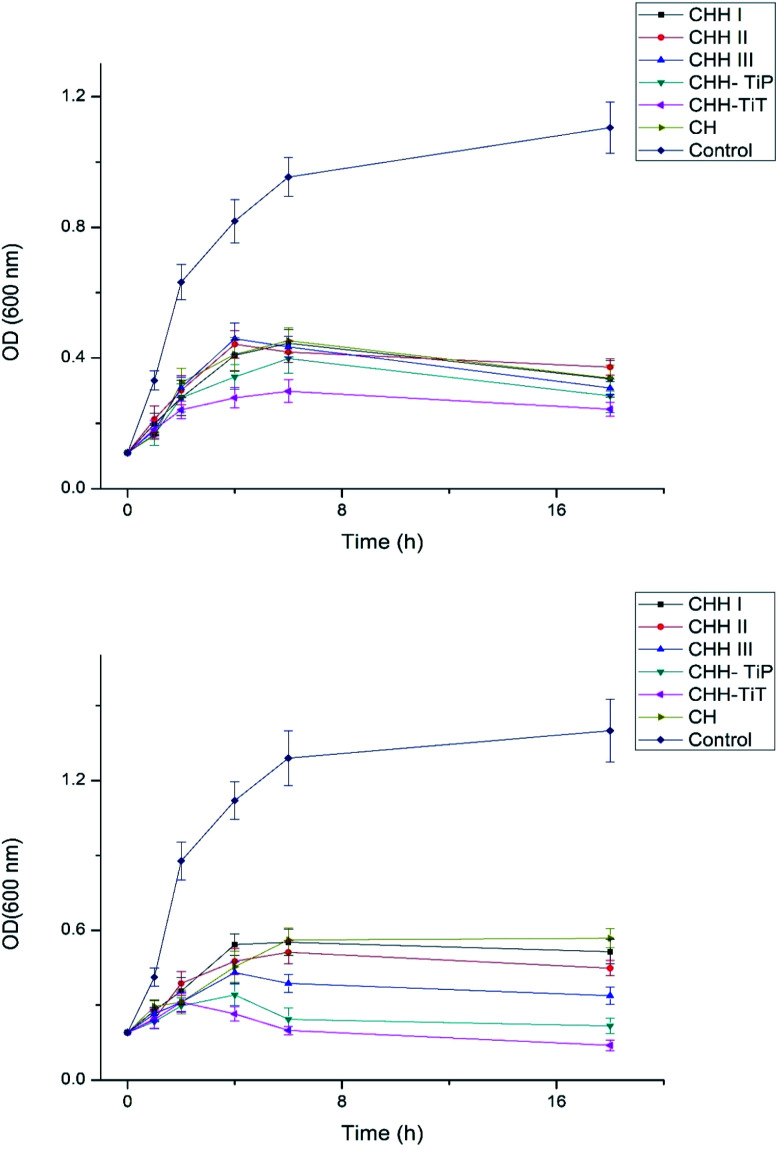
Antibacterial studies of CHH I–III, CHH-TiP, CHH-TiT and CH with *E. coli* (top) and *S. aureus* (bottom). Data here is represented as means of triplicate values ± SD.

From SI 3.11 in ESI,† it is observed that CHH I had the highest water uptake followed by CHH II and then CHH III. This is due to the increasing content of HNTs in the membranes, the HNTs interact with the chitosan polymer, resulting in a decreased number of free hydroxyl groups in CTS; the water molecules have lesser sites available for hydrogen bonding with the chitosan matrix.^32,33^ The pH of CHH I–III (SI 3.12 in ESI†) in the saline started at a mildly acidic environment (6.56–6.70) on day 1 and developed a mildly basic (7.27–7.40) environment by day 14, possibly due to the presence of n-HAP (Lewis base). No shape change (morphological index) have been reported in RBCs in the pH range of 6.5 to 8.0, so it is assumed that no detrimental effects are caused to the cells or their cellular functions in the initial few days when the pH of the samples is in the slightly acidic range of 6.56–6.93.^34^ The hemolytic behavior of the implants is a very common complication that arises due to the contact between the medical implant and the human blood. Acute hemolysis leads to the release of hemoglobin and organ failure in the host. The compatibility of CHH I–III membranes with the human erythrocytes was established using hemolytic assay (Fig. 5). CHH I–III membranes had hemolysis percentages less than 5%, so it is concluded that CHH I–III can be safely applied for *in vivo* purposes without causing acute hemolysis.^35^ The results support a similar finding that a low concentration of HNTs has no toxic effect on human erythrocytes.^36^ The current grafting procedures commonly suffer from the post-surgical infections in the region surrounding the implant, so there is a clinical urgency to engineer membranes that can fight the infections on their own. This would eliminate the need for secondary surgery to sterilize the region or to remove the implant. From Fig. 6, it is observed that the bacterial growth has been significantly inhibited in both the bacterial suspensions with the addition of CHH I–III nanocomposites. In both the suspensions, the reduction in bacterial growth is higher for nanocomposites with higher HNT loading. Amongst the different *S. aureus* suspensions, the cell growth started to stall in the suspension containing the CHH III membrane after 4 h. The cell growth came to a halt after 6 h in the *S. aureus* suspension. These results establish that a higher concentration of HNTs leads to a higher antibacterial efficacy in these membranes. The bacterial inhibition is slightly more in *S. aureus* suspension than in the *E. coli* suspension; this is attributed to the presence of an additional outer liposaccharide membrane in Gram-negative microbes (*E. coli*), leading to the increase in their resistance to antibiotics and antibacterial agents.^37^

**Fig. 7:**
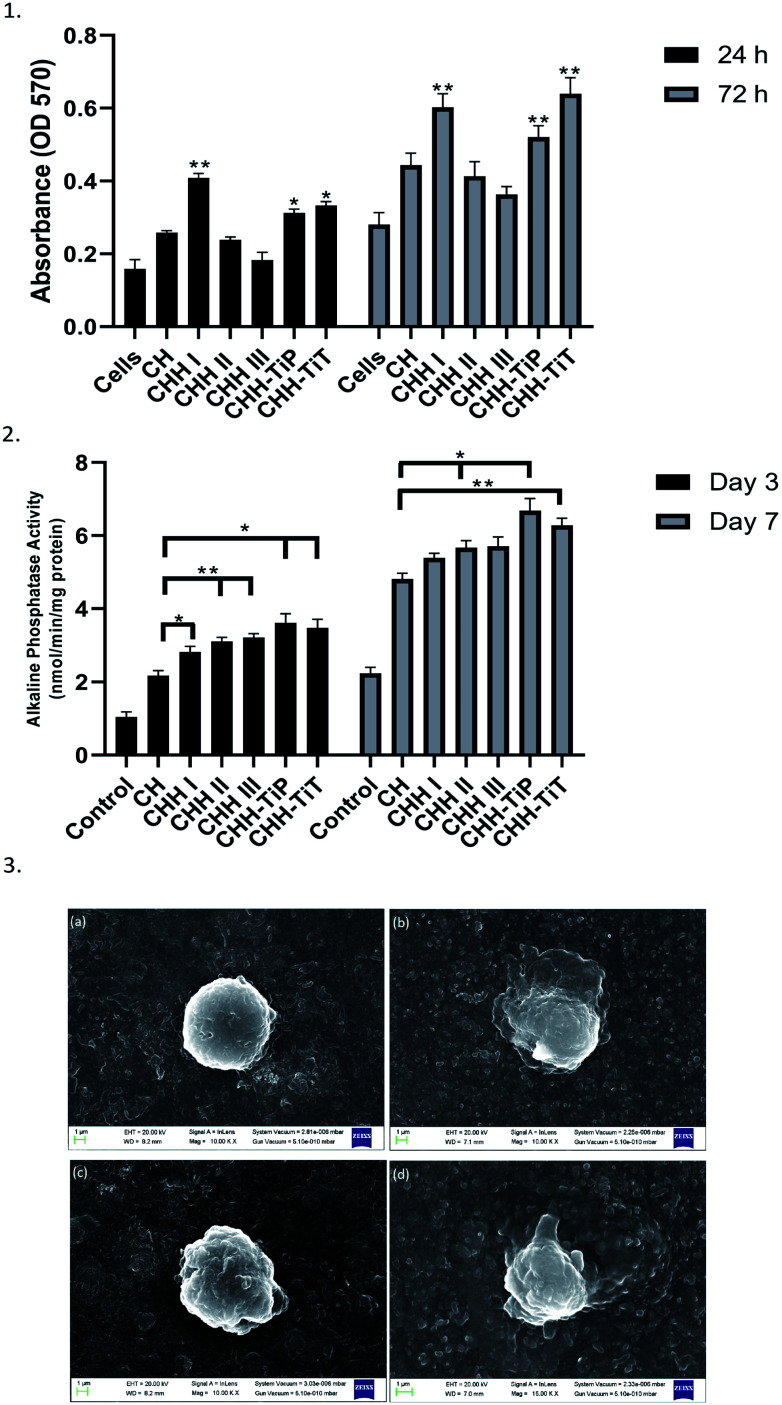
(1) Absorbance values from the MTT assay to quantify the MG-63 cell proliferation on the surface of the membranes. (2) ALP activity of the cells seeded on the membranes on day 3 and 7. (3) FESEM images of attached MG-63 cells on CHH I after 24 h (a), 72 h (b) and CHH-TiT after 24 h (c), 72 h (d). Data in (1) and (2) is represented as means of triplicate wells ± SD, wherein * indicates *p* < 0.05 and ** indicates *p* < 0.01 when compared with CH membrane at respective time points.

From Fig. 7(1), it is observed that the cell proliferation after 24 h and 72 h was maximum on CHH I (5% HNT) amongst CHH I–III and decreased with increasing HNT loading. CHH I–III contain a high amount (>80%) of biocompatible CTS.^7^ The cell proliferation was higher on CH II and CHH III (CTS/n-HAP) than the control well but lower than the CH membrane. This indicates that cell growth is dependent on HNT concentration. The highest cell growth is observed for the lowest (5%) HNT loading and relatively lesser cell proliferation is observed for higher HNT loading. Since the cell proliferation at CHH I–III is higher than that in the control well, it can be concluded that CHH I–III have an overall positive effect on the proliferation of human osteoblast-like MG-63 cells. From SI 3.14 in ESI,† it can be observed that the addition of HNTs in CHH I–III leads to the release of Si and Al ions in the buffer solution, this increased release of Al ion in the cell media could have contributed to the decreased cell proliferation in CHH II and CHH III, where the ion release concentration is higher in comparison to CHH I. Hallab *et al.* (2002) have reported that the metal ions such as Al reduce the osteoblast proliferation.^38^

The attachment of the osteoblast-like cells on the surface of the membrane depends on the surface topography and chemistry. From the FESEM image (Fig. 7(3a and b)), it can be observed that the cell attachment improved over the span of seeding time on the membrane. On the 3^rd^ day after seeding, the cells started to attach and spread on the membrane surface by forming a mineralized layer. The cells displayed an elongated polygonal like shape with outward extending filopodia in contact with the membrane. These results confirm the successful attachment of the MG-63 cells on the surface of CHH I.

ALP enzyme activity is a marker of osteoblastic differentiation and mineralization for bone regeneration, an increased ALP activity signifies maturation of the extracellular matrix. From our studies on the ALP expression of the MG-63 cells on the CHH I–III membranes, we observed that the CHH I–III membranes have a higher ALP activity on the 3^rd^ and 7^th^ day after the initial seeding than the CH membranes. The increased activity can be attributed to the presence of HNTs in CHH I–III. From similar studies on composites, the presence of Si at the surface of HNTs is expected to upregulate the secretion of collagen I^39^ and bioactivity of hydroxyapatite particles in the membrane.^40^ The overall trend in the ALP activity at day 3 to day 7 supports the claim that there is an increase in osteogenic potential of CHH I–III over CH, possibly due to the addition of HNTs.

From the above results, it was established that CHH II (7.5% HNT) had the most favorable mechanical properties such as UTS and elasticity. But, CHH III (10% HNT) had higher antibacterial efficacy than CHH II and the MG-63 cell proliferation on CHH II was lower than CHH I (5% HNT) and the control sample (CTS/n-HAP). To overcome the shortcomings in the biological characteristics of the CHH II membrane, we separately added TiO_2_ NP (0.2%) (CHH-TiP) and TiO_2_ NT (0.2%) (CHH-TiT) into polymer constructs with a similar composition as that of CHH II to synthesize new membranes. The aim of this second study was to augment the favorability of the biological characteristics of the membrane such as anti-bacterial efficacy, osteoblast-like cell proliferation, and differentiation potential, at the optimized HNT loading (7.5%). We then studied the mechanical and biological properties of these new membranes (CHH-TiP and CHH-TiT; Table 1) and compared them with each other.

### 3.2 Comparative study between TiO_2_ NP and TiO_2_ NT doped CTS/n-HAP/HNT matrix with 7.5% HNTs

From SI 3.2 in ESI,† small peaks at 2*θ* = 37.85° and 37.84° can be observed in the XRD patterns, corresponding to the presence of TiO_2_ NP (SI 3.3 in ESI†) and TiO_2_ NT (SI 3.3 in ESI†) in CHH-TiP and CHH-TiT respectively. The small size of the peaks is due to the high dispersion of a small amount (0.2%; w/w) of these nanoparticles in the CTS/n-HAP/HNT polymer matrix. In addition, other peaks can be observed at 2*θ* = 25.36°, 47.98° and 62.81° for both TiO_2_ NP and NT in SI 3.2 in ESI,† which are in good accordance with the standard ICDD (JCPDS) card # 21–1272 (anatase TiO_2_).

The presence of TiO_2_ NP and TiO_2_ NT in CHH-TiP and CHH-TiT respectively can also be confirmed through the presence of peaks at 1372 cm^−1^ in their respective FTIR spectra (SI 3.4 in ESI†).

The TiO_2_ NP (SI 3.7 in ESI†) are spherical in shape with the particle diameters in the range of 30–35 nm. From the TEM (Fig. 3(d)) and FESEM images (SI 3.8 in ESI†) of TiO_2_ NT, it can be inferred that the synthesized entities are tube-shaped with an average length of the order of 300 nm and diameter in the range of 30–70 nm. The crystallinity of the synthesized TiO_2_ nanotubes is manifested by the highly resolved TiO_2_ lattice fringe details in the TEM image (Fig. 3(d)).

In CHH-TiP, the ultimate tensile strength (UTS) increased over the UTS of CHH II due to the integration of TiO_2_ NP (0.2%) in the polymer matrix (Table 2). An even higher increase in the ultimate strength was observed in CHH-TiT over CHH II due to the addition of TiO_2_ NT (0.2%), as the tubular structure of the TiO_2_ NT facilitates the load transfer from polymer matrix and other nanofillers to the additional TiO_2_ nanotubes integrated into the nanocomposite. The trabecular bone is reported to have a tensile strength of 50 MPa,^21^ so CHH II (58.91 MPa), CHH-TiP (64.36 MPa) and CHH-TiT (67.14 MPa) showcasing an ultimate tensile strength greater than 50 MPa, can serve as potential bone tissue regeneration templates without undergoing any potential mechanical failure. These UTS values are significantly higher in comparison to the recently reported CTS based nanocomposite membranes.^7,22,23,27,41,42^

From the results of TGA, the effect of TiO_2_ NP and TiO_2_ NT addition on the thermal resistance of the chitosan matrix containing 4.8% n-HAP and 7.5% (w/w) HNTs was studied (SI 2 in ESI†). CHH-TiT had a higher residual weight at all the heating temperatures and had a protracted degradation curve over CHH-TiP and CHH II (SI 3.11 in ESI†). It can be easily concluded that the addition of TiO_2_ NT had a higher increase in thermal resistance over TiO_2_ NP addition in the CTS/n-HAP/HNT polymer matrix. From Fig. 4, it is observed that CHH-TiT has the highest storage modulus at all frequencies followed by CHH-TiP and CHH I; this is attributed to the presence of TiO_2_ NT and TiO_2_ NP along with 7.5% HNTs. TiO_2_ NT has a high aspect ratio, thus provides a higher potential for enhancement in elasticity than TiO_2_ NP to the CTS/n-HAP/HNT network. The trend of *E*′ and *E*′′ confirm the viscoelastic nature of both the CHH-TiT and CHH-TiP.

The results of the hemolytic assay (Fig. 5) established the compatibility of CHH-TiT and CHH-TiP with the human blood as the hemolysis (%) was low (<5%) for both these nanocomposite membranes. These results indicate that the addition of TiO_2_ NP and TiO_2_ NT in small amounts doesn't have any toxic effects on human erythrocytes. These values are significantly lower than those reported in similar studies.^23,41^

**Fig. 8:**
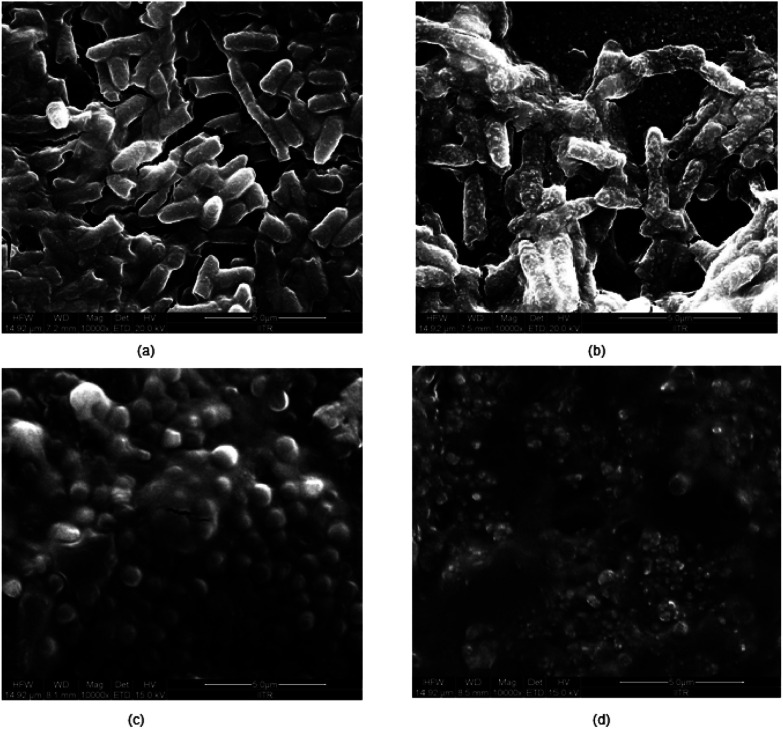
FESEM images of untreated *E. coli* (a), CHH-TiT treated *E. coli* (b), untreated *S. aureus* (c) and CHH-TiT treated *S. aureus* (d).

From the Fig. 6, it is established that CHH-TiT and CHH-TiP had a significantly higher antibacterial efficacy than CHH II (7.5% HNT) and CHH III (10% HNT), which is attributed to the addition of TiO_2_ NT and TiO_2_ NP respectively. The final OD for CHH-TiT in *S. aureus* suspension at 18 h was even lower than the initial OD (at 0 h), indicating superior anti-bacterial properties afforded by TiO_2_ NT addition. From Fig. 8(b), it was observed that the nanoparticles leached from CHH-TiT membranes were internalized within the rod-shaped body of *E. coli* and small bleb-like structures could be observed on their surfaces. The rod-like shape of *E. coli* seems to be compromised and bacteria cells didn't possess the same structural integrity as their untreated counterpart (Fig. 8(a)). Spherical shaped bacterial inner vacuoles also appeared in the treated *E. coli* sample. From Fig. 8(d), it was observed that the microbes shriveled and deformed upon treatment with CHH-TiT, signaling necrosis of *S. aureus*. These results show the superior anti-bacterial efficacy of the CHH-TiT film.

Upon the degradation of the membranes, the TiO_2_ nanoparticles could potentially accumulate in the spleen, kidney or liver. But the amount of TiO_2_ present in a CHH-TiT or CHH-TiP membrane for healing a 2–4 cm long segmental bone defect is very minimal (0.3 mg), which is well below the safety limit of 2.25 mg TiO_2_ NPs per kg bw per day.^43^

From Fig. 7(1), it is established that CHH-TiT had a higher osteoblast-like cell proliferation than CHH-TiP and CHH II, even at the same HNT and n-HAP loading. This is due to the highly osteoconductive nature of Titania tubes.^18^ The increased cell proliferation on CHH-TiT and CHH-TiP in comparison with CHH II proves that the respective addition of TiO_2_ nanoparticles and nanotubes in lower quantities (0.2%) increased the cell proliferation of osteoblast-like MG-63 cells on their respective surfaces. The cell attachment improved on the surface of CHH-TiT from day 1 to day 3 (Fig. 7(3c and d)) as the cells at day 3 have a relatively flatter morphology with filopodia extending outwards from their bodies over the membrane surface. Moreover, the ALP activity (Fig. 7(2)) is higher on CHH-TiP and CHH-TiT membranes than the CHH I–III membranes on day 3 and day 7. This is attributed to the presence of TiO_2_ in CHH-TiP and CHH-TiT, which stimulates ALP activity and hence would increase the *in vivo* osteoblast differentiation as reported in a similar study.^44^ These results support the osteogenic potential of CHH-TiP and CHH-TiT membranes, but further investigations on the osteogenic differentiation at the molecular and cellular levels with the hMSCs are required. Our future studies will be focused on the osteogenic differentiation of hMSCs and *in vivo* investigations of the CHH-TiT membrane implantation.

Recently, Zheng *et al.*, 2019 (ref. 47) have reported the preparation of HNT-HAP hybrid *via* facial hydrothermal reaction process. Their synthesized CTS/HNT-HAP composite has the ability to enhance cytocompatibility and osteogenic differentiation ability towards preosteoblasts. In addition, Jing *et al.*, 2017 (ref. 48), have reported that the filler content of n-HAP and HNT can be varied and optimized in a poly(caprolactone) (PCL) matrix to enhance the cellular differentiation on its surface. Valverde *et al.*, 2019 (ref. 49) have reported that the implants obtained from the addition of hyaluronic acid/chitosan multi-layer films on the Ti-6Al-4V alloys enhance their anti-bacterial efficacy. The CHH-TiT membrane can also be considered for use with permanent implants after further investigations.

Our dual-study has established that the addition of TiO_2_ NT (0.2%) to CTS/n-HAP/HNT nanocomposite at the optimized HNT loading of 7.5% results in a nanocomposite (CHH-TiT) with superior mechanical and *in vitro* biological properties. CHH-TiT can be used to mimic the natural extracellular matrix of the bone as it possesses the required multi-functionalities.^7,8^ Moreover, the use of CHH-TiT as a food packaging membrane can be further investigated as it is biocompatible and has superior antimicrobial efficacy. Our future work will further investigate the *in vitro* and *in vivo* effects of these multi-functional membranes on human bone regeneration. These nanocomposite membranes have the potential to be employed for healing short segmental bone defects (2–6 cm) and the defects with limited contact. The membranes can be wrapped around the defect using bioabsorbable screws^45^ as it can withstand the mechanical load and provide a surface for osteoblast attachment, proliferation, and differentiation. The mechanical rigidity of the CHH-TiT membrane would provide a barrier for maintaining the original shape of the bone and avert any postoperative attachment between the bone and the surrounding soft tissues. The CHH-TiT membrane has the potential to induce rapid necrosis in the bacterial cells, hence it can also be used for treating the pre-infected bone defect models by swathing the membrane around the infected defect.

## 4. Conclusion

In the first stage of the study, we successfully fabricated CTS/n-HAP/HNT (CHH I–III) nanocomposites which are yet to be reported in the literature. The primary aim was to optimize the HNT loading in CHH I–III so that the properties of the membrane with optimized loading can mimic the mechanical and biological properties of the natural bone extracellular matrix for successful bone tissue regeneration. FESEM and TEM images confirmed the uniform dispersion of HNTs in CHH I (5% HNT) and CHH II (7.5% HNT), while the presence of larger agglomerates in CHH III (10% HNT) indicated increased tube–tube interactions at 10% HNT loading. TEM images also confirmed electrostatic attractions between the CTS matrix and the dispersed HNTs. AFM images revealed the presence of nano-dimensional pores (200–300 nm) on the surface of CHH I–III. The nanocomposite with 7.5% HNT loading (CHH II) displayed the highest tensile strength of 58.91 MPa and a highly viscoelastic nature. But, the proliferation of osteoblast-like MG-63 cells on CHH II was comparatively lower in comparison to CHH I (5% HNT) and CH (control sample; CTS/n-HAP) and the antibacterial efficacy was higher at 10% HNT loading (CHH III). The ALP activity was higher for CHH I–III in comparison with CH membranes due to the presence of Si at the surface of HNTs in CHH I–III. With an aim to enhance the biological properties such as cell proliferation and antibacterial efficacy of CTS/n-HAP/HNT nanocomposite at 7.5% HNT loading, we separately doped TiO_2_ nanoparticles and TiO_2_ nanotubes into CHH II like polymer formulations. The ultimate tensile strength of CHH-TiP and CHH-TiT increased to 64.35 MPa and 67.14 MPa respectively, due to TiO_2_ NP and NT addition. These rigid membranes will help in restricting the growth of the healing bone to a specific region and avert any postoperative attachment between the bone and the surrounding soft tissues. CHH-TiT had the highest elasticity amongst all the membranes and had an increased thermal resistance over CHH-TiP and CHH II. CHH-TiT (7.5% HNT, 0.2% TiO_2_ NT) followed by CHH-TiP (7.5% HNT, 0.2% TiO_2_ NT), considerably reduced the microbial growth (both Gram-positive and Gram-negative) by initiating cell blabbering and subsequent necrosis, their efficacy was even higher than CHH III (10% HNT). All the nanocomposites had hemolysis (%) well below the limit of acute hemolysis, thus establishing their compatibility with the human erythrocytes. The cell proliferation of osteoblast-like MG-63 cells was maximum for CHH I (5% HNT) while for the membranes with 7.5% HNT loading, it increased well above the cell proliferation at CH with the addition of TiO_2_ nanotubes (CHH-TiT) and nanoparticles (CHH-TiP) to the membrane. The expression of differentiation marker for the osteoblast-like MG-63 cells increased over time and it was maximum for CHH-TiP and CHH-TiT owing to the presence of 7.5% HNTs and 0.2% TiO_2_. These results establish the potential use of CHH-TiT in bone tissue regeneration, but further *in vitro* and *in vivo* studies are required before it can be used in clinical settings. This study highlights a feasible two-staged strategy to engineer bioresorbable membranes possessing enhanced anti-bacterial efficacy to inhibit any post-implantation bacterial infections and simultaneously serve as a template for guided bone tissue regeneration.

## Conflicts of interest

All the authors have no competing interests to disclose.

## Supplementary Material

RA-009-C9RA08366A-s001
