# Supplementary material for: TiO2 doped chitosan/hydroxyapatite/halloysite nanotube membranes with enhanced mechanical properties and osteoblast-like cell response for application in bone tissue engineering
Source: RSC Adv. 2019 Dec 2;9(68):39768–79. doi: 10.1039/c9ra08366a (PMC9076061; doi:10.1039/c9ra08366a)
Supplement: RA-009-C9RA08366A-s001 [file RA-009-C9RA08366A-s001.pdf]

Electronic Supplementary Material (ESI) for RSC Advances.
This journal is © The Royal Society of Chemistry 2019

Supporting Information

TiO_2 doped Chitosan/ Hydroxyapatite/ Halloysite Nanotube membranes with enhanced mechanical properties and osteoblast-like cell response for application in Bone Tissue Engineering

Sarim Khan [a,c], Viney Kumar [b], Partha Roy [b], Patit Paban Kundu [a,*]

[a] Department of Chemical Engineering, Indian Institute of Technology, Roorkee 247667, India
[b] Department of Biotechnology, Indian Institute of Technology, Roorkee 247667, India
[c] Institute for Medical Engineering and Science, Massachusetts Institute of Technology, Cambridge, USA

* Corresponding Author
Email: ppkfch@iitr.ac.in (Patit Paban Kundu), sarimk@mit.edu (Sarim Khan)

Supplementary materials:

SI 1.1
Water Absorption Study

At first, the dry weights (W_0) of CHH I-III, CHH-TiP, CHH-TiT and CH were measured. Thereafter the samples were immersed in distilled water for up to 24 hrs. The samples were then displaced periodically from the water and gently blotted with filter paper to remove water adsorbed at the surface. The samples were then weighed again (W_t), after 1, 2, 3, 4, 6, 12 and 24 h of first immersing them into the water.

SI 1.2
Hemolytic assay

Following approval from the Institute human ethics committee of IIT Roorkee and with due consent, blood was collected from 3 adult individuals. The lysis of the RBC leads to the release of hemoglobin and subsequent centrifugation separates other cell debris and the intact cells. The amount of hemoglobin in the supernatant depends on the number of cells lysed by the respective sample solutions of CHH I-III, CHH-TiP, CHH-TiT and CH. The freshly collected human blood sample was centrifuged at 3000 rpm and 4°C, to separate the blood into erythrocytes (RBC), plasma and Buffy Coat. The RBC's were then collected at the bottom of the centrifuge tube and were then subsequently washed with phosphate buffer solutions (PBS) at pH 7.4 and thereafter re-suspended in the same buffer. Then, 50 µl of each sample solution was taken in a centrifuge tube, that was then subsequently diluted with an additional 900 µl of PBS and finally, 50 µl of RBC sample was added to the above tube. The tubes were then incubated in dark for 10 minutes at 4°C and were then subsequently centrifuged. The optical density (OD) of the supernatant following the centrifugation was measured at 540 nm using a spectrophotometer. The Positive control sample (50 µl RBC + 950 µl H_2O) and Negative control sample (50 µl RBC + 950 µl PBS) were also prepared to compare their OD values with the OD of the supernatant of the respective sample solutions.

SI 1.3
MTT assay

The culture was maintained using Alpha Minimum Essential Medium (α-MEM) (Sigma Aldrich). The medium was replaced at regular intervals. To carry out the MTT assay, 1 mg of each sample was maintained in polystyrene 96-well sterilized plates. 5X103 cells were seeded on each scaffold (experimental) and wells without scaffolds (experimental control) in 200 µL of the medium. The cells were incubated at 37°C for 24 h in a humidified incubator with 5% CO_2. Subsequently, 20 µL of 3-(4,5-dimethylthiazol-2-yl)-2,5-diphenyltetrazolium bromide (MTT) (Sigma Aldrich) dissolved in 1X PBS (5mg/mL) was added to each well (experimental variables and experimental control) followed by a 4 h incubation at 37°C. Thereafter, the MTT containing medium was replaced from each well with 200 µL of

dimethyl sulfoxide (DMSO), to dissolve the formazan crystals. Finally, the absorbance of the solution was recorded at 570 nm using a Fluostar Optima plate reader (Germany).

SI 1.4
Antibacterial efficacy study

Both the microbes were procured from NCCS, Pune. In outline, E. coli was inoculated in Luria-Bertani broth (LB) and S. aureus was inoculated in Nutrient Broth (NB), for the exponential growth of the microbes. 0.03 mg of each film was added into 30 mL of bacterial suspension and subsequently incubated at 37°C in a vibrator at 180 rpm for 18 h. 300 microliter of suspension aliquots were taken out from each suspension at time intervals of 1, 2, 4, 6, 18 h to measure their optical density (OD) at 600 nm. An increase in OD signifies bacterial growth in the suspension. To examine the bacterial structure of E. coli and S. aureus before and after treating them with CHH-TiT film we drop-fixed the respective suspensions on a square glass film, which were subsequently taken for imaging on a Field Emission Scanning Electron Microscope (FE-SEM Quanta 200 FEG).

SI 1.5
Alkaline Phosphatase (ALP) activity test

The MG-63 cells were seeded on the films for 3 or 7 days in a humidified incubator at 37°C with 5% CO_2. The cells were then lysed using 500.0 µL of lysis solution (0.2% TritonX-100 (w/v)). 50 µL of supernatant from lysis was incubated along with 100 µL of pNPP substrate solution for ELISA (Sigma-Aldrich) at 25°C. Then the absorbance reading of the wells were taken using a microplate reader at 405 nm after ageing for 30 minutes. ALP activity of the cells cultured without any films served as the control. The ALP activity values were normalized by the amount of protein present in the well. The protein was quantified using Bicinchoninic acid (BCA) protein assay kit (Sigma-Aldrich). Briefly, 50 µL of cell lysate supernatant was added to 200 µL of the BCA reagent, followed by incubation at 37°C for 30 min. The optical absorbance was measured for each well at 562 nm using a microplate reader to quantify the protein. Finally, the Protein content and ALP activity were evaluated using the standard curve derived from the serial dilutions of the standard reagents.

SI 2

Table SI 2.1: TGA results for CHH II, CHH-TiP, CHH-TiT, and CH.

	CHH II	CHH-TiP	CHH-TiT	CH
Temperature(in °C) at 20 % Weight Loss	219.24	227.54	250.12	163.91
Residue Weight(%) at 300°C	58.34%	57.84%	61.77%	52.22%
Residue Weight(%) at 600°C	36.24%	36.13%	39.01%	29.69%

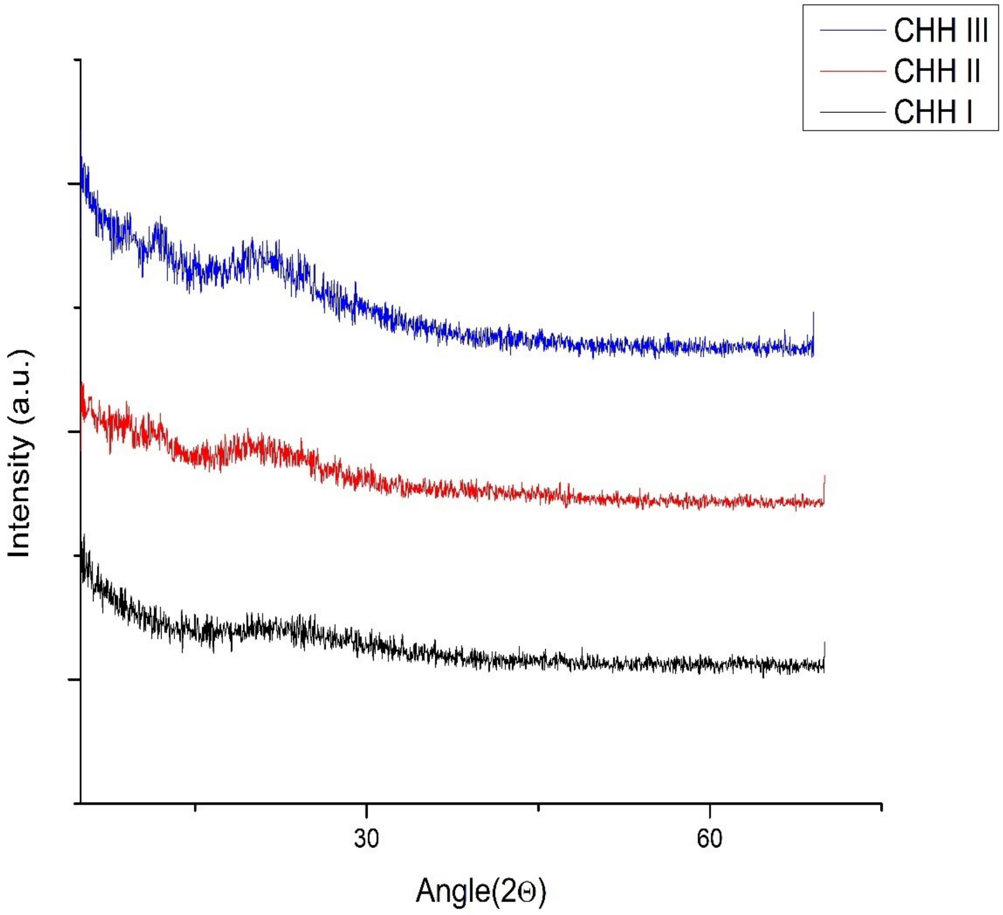

SI 3.1: XRD patterns of CHH I-III

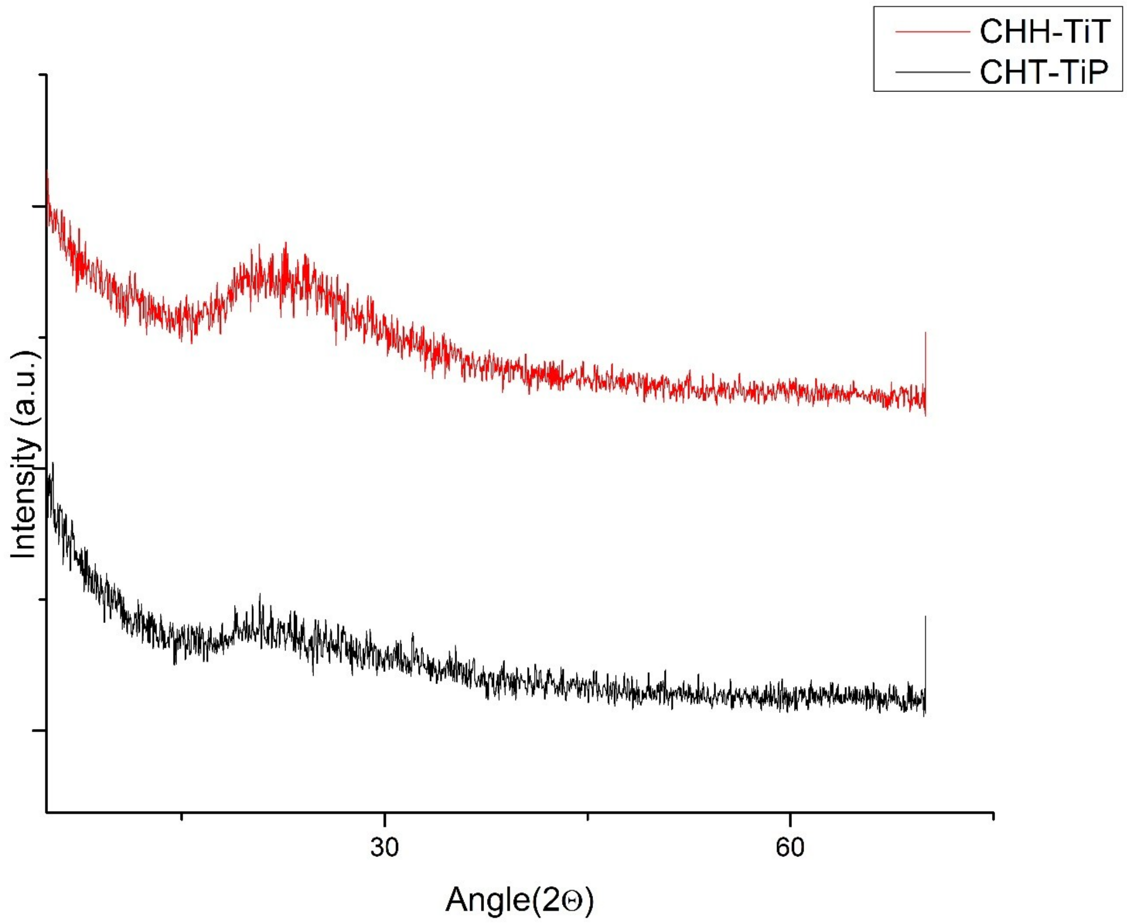

SI 3.2: XRD patterns of CHH-TiP and CHH-TiT

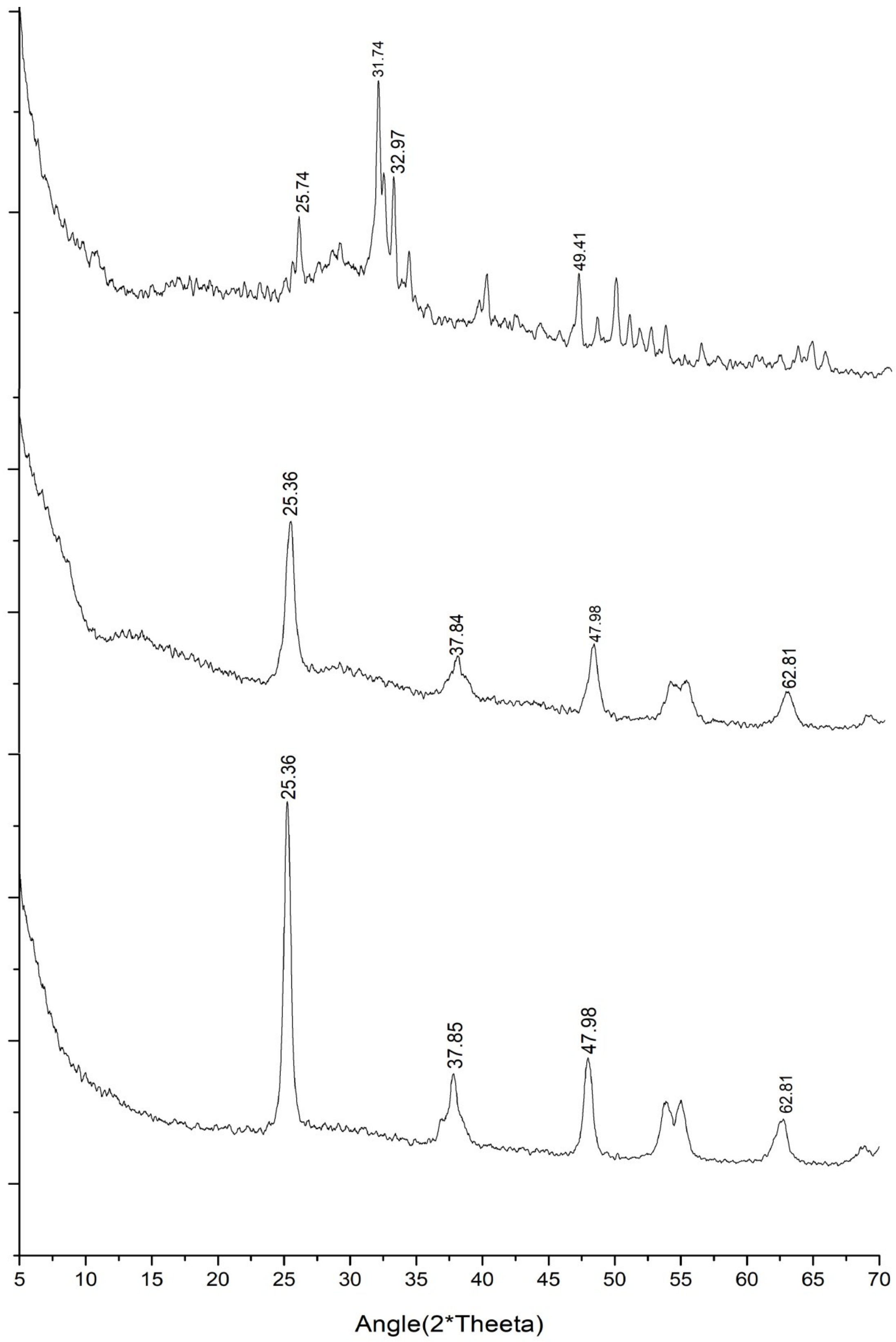

SI 3.3: XRD patterns of HAP, TiO_2 NP and TiO_2 NT (From top to bottom in order).

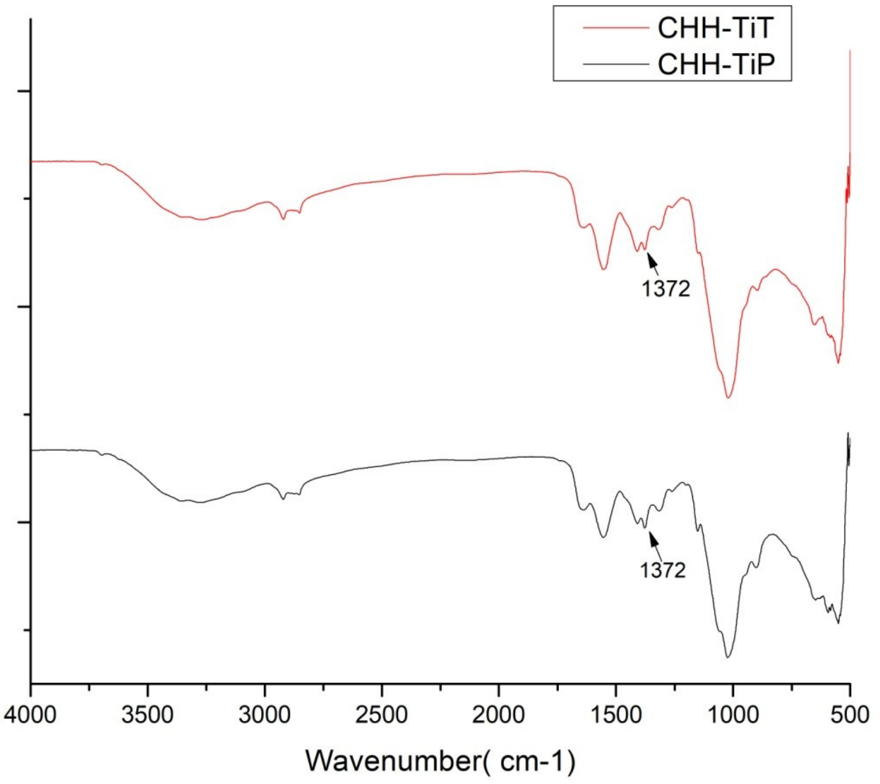

SI 3.4: FTIR spectrum of CHH-TiP and CHH-TiT

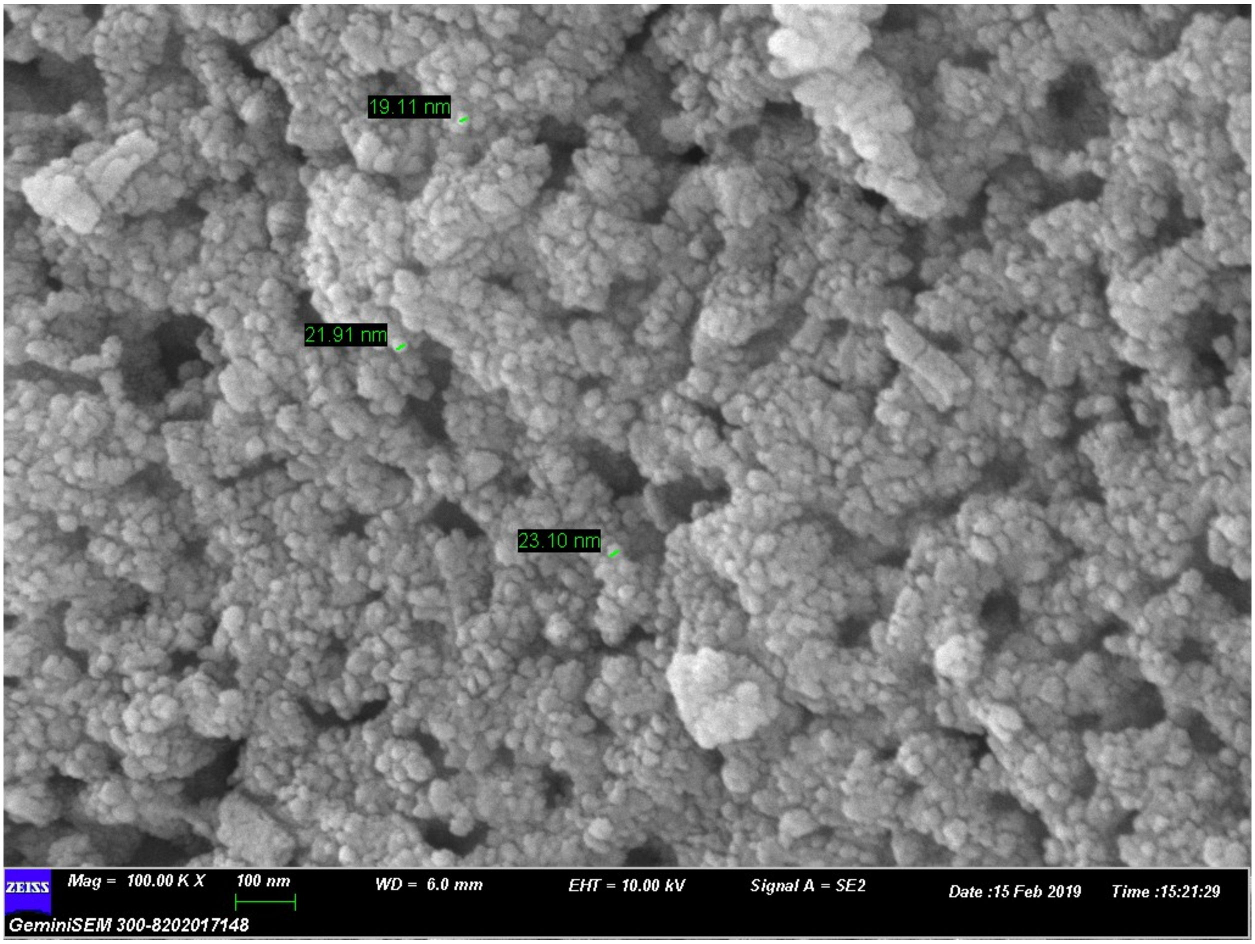

SI 3.5: FESEM Image of n-HAP Nanoparticles

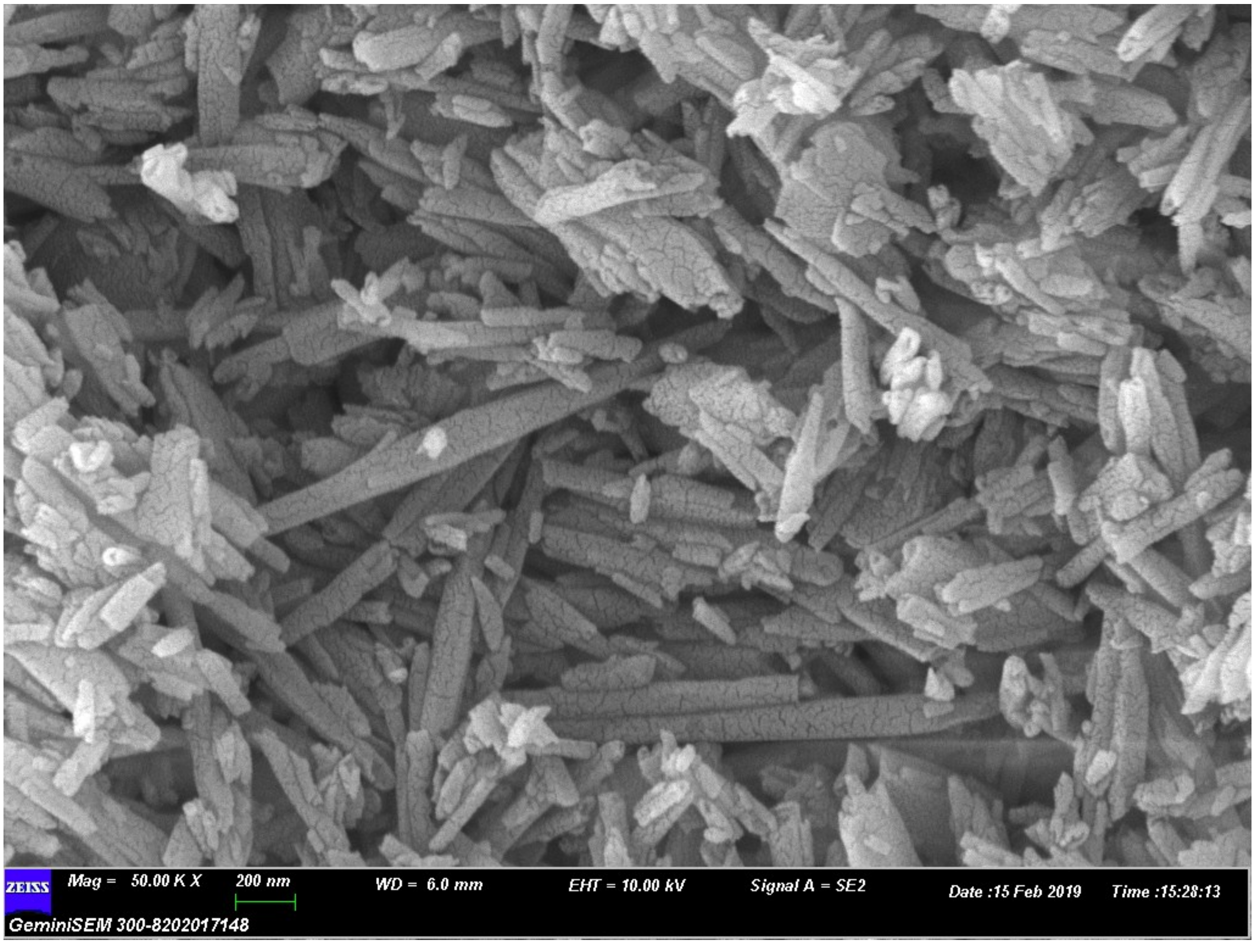

SI 3.6: FESEM Image of Halloysite Nanotubes

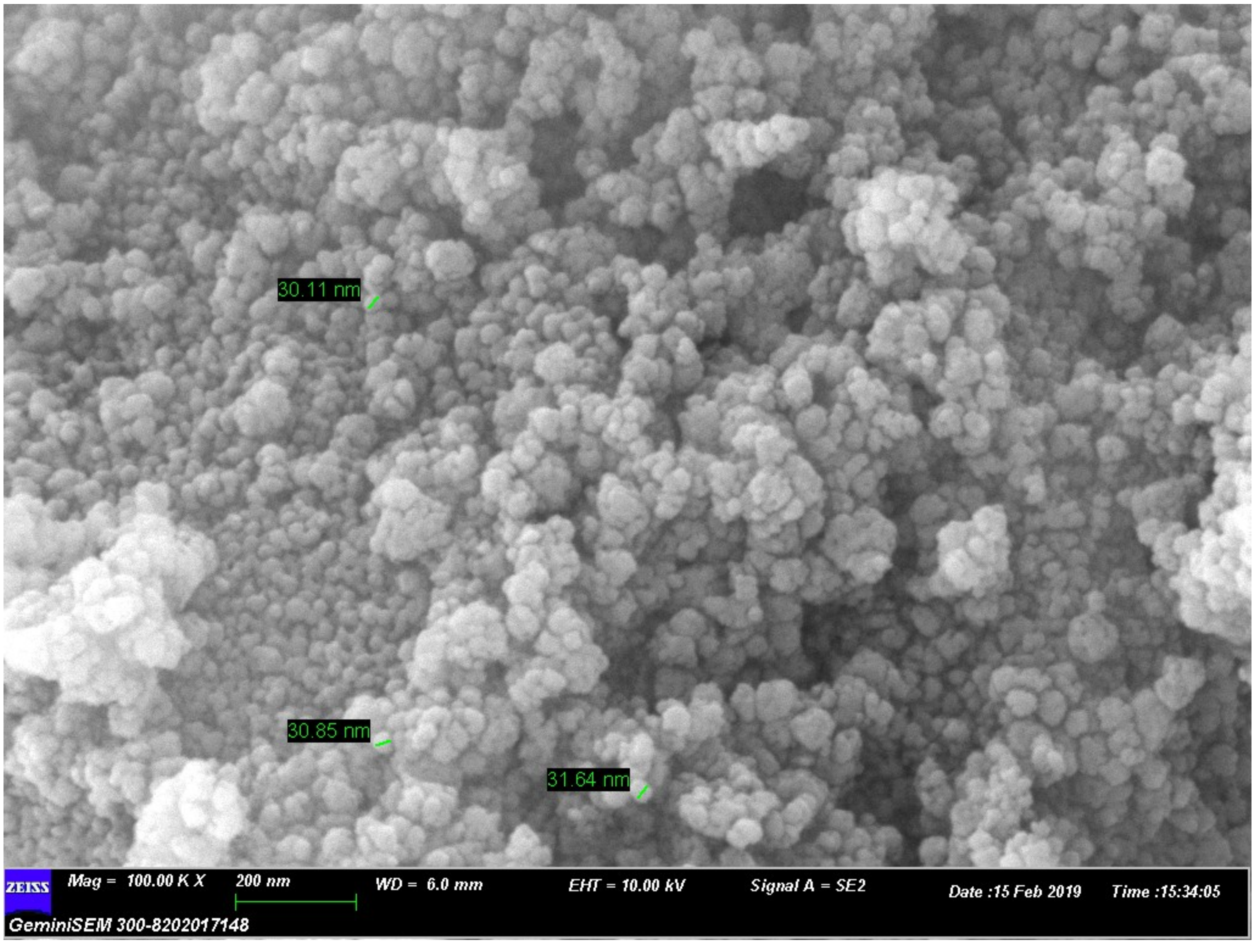

SI 3.7: FESEM Image of TiO_2 Nanoparticles

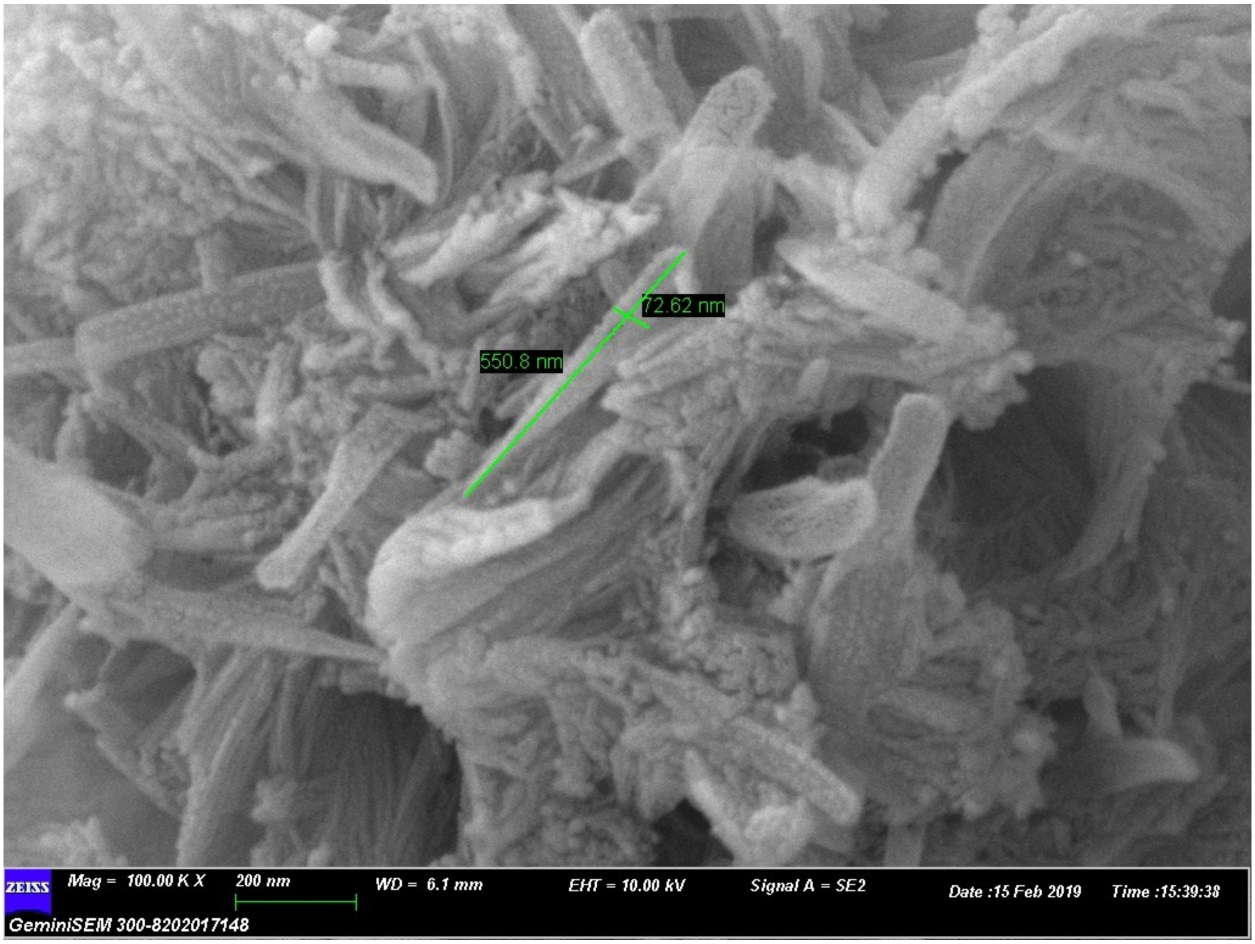

SI 3.8: FESEM Image of TiO_2 NT

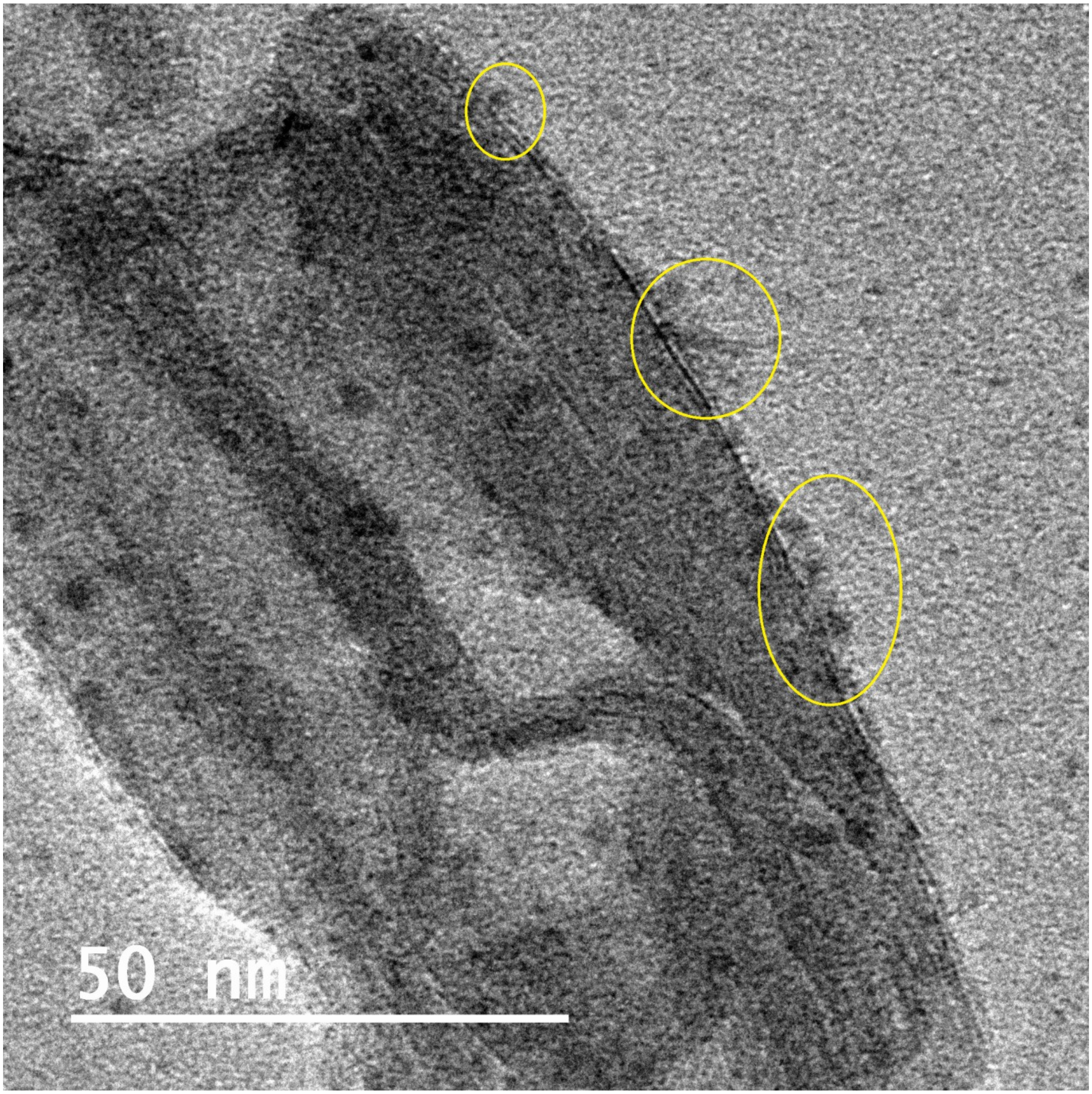

SI 3.9: TEM Image of HNT in CHH II

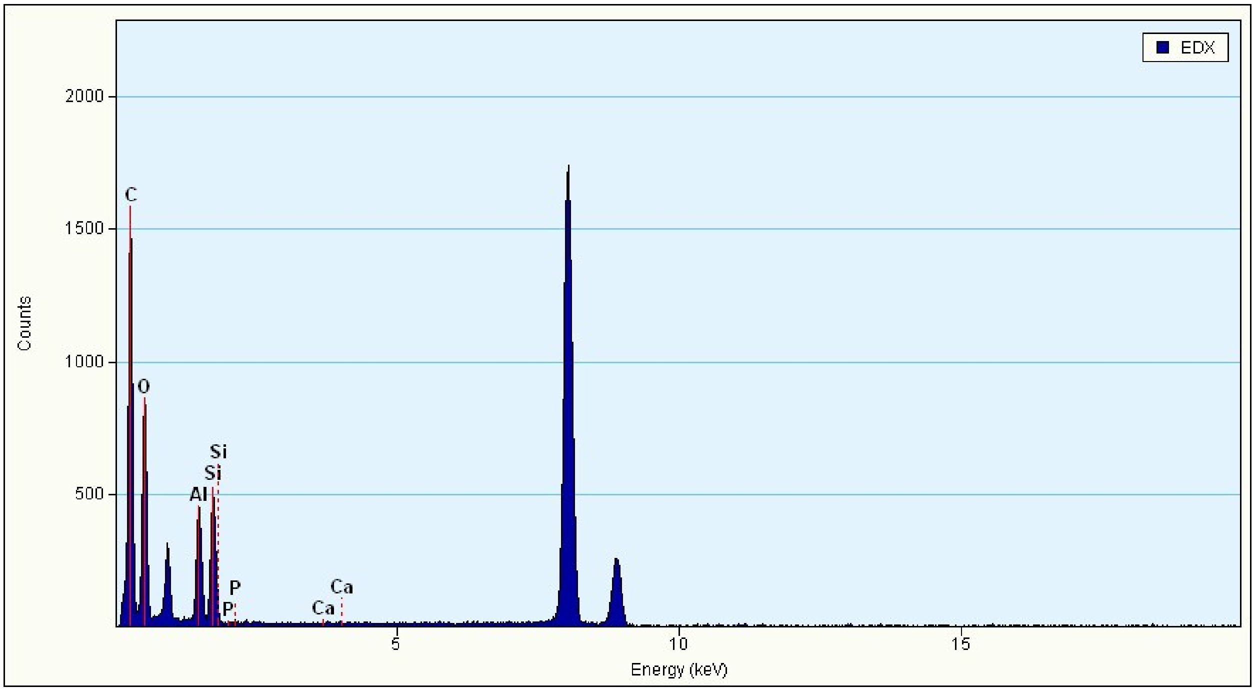

SI 3.10: EDX of CHH II

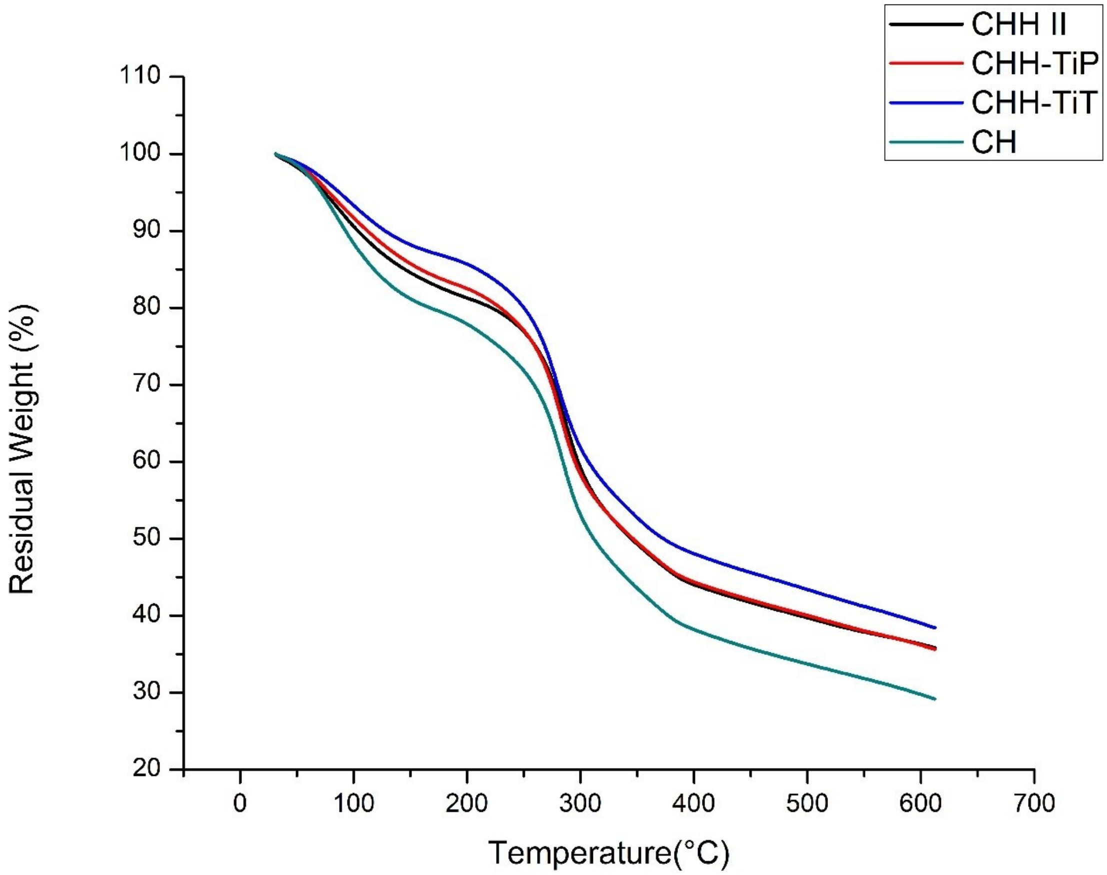

SI 3.11: TGA of CHH II, CHH-TiP, CHH-TiT and CH with temperature.

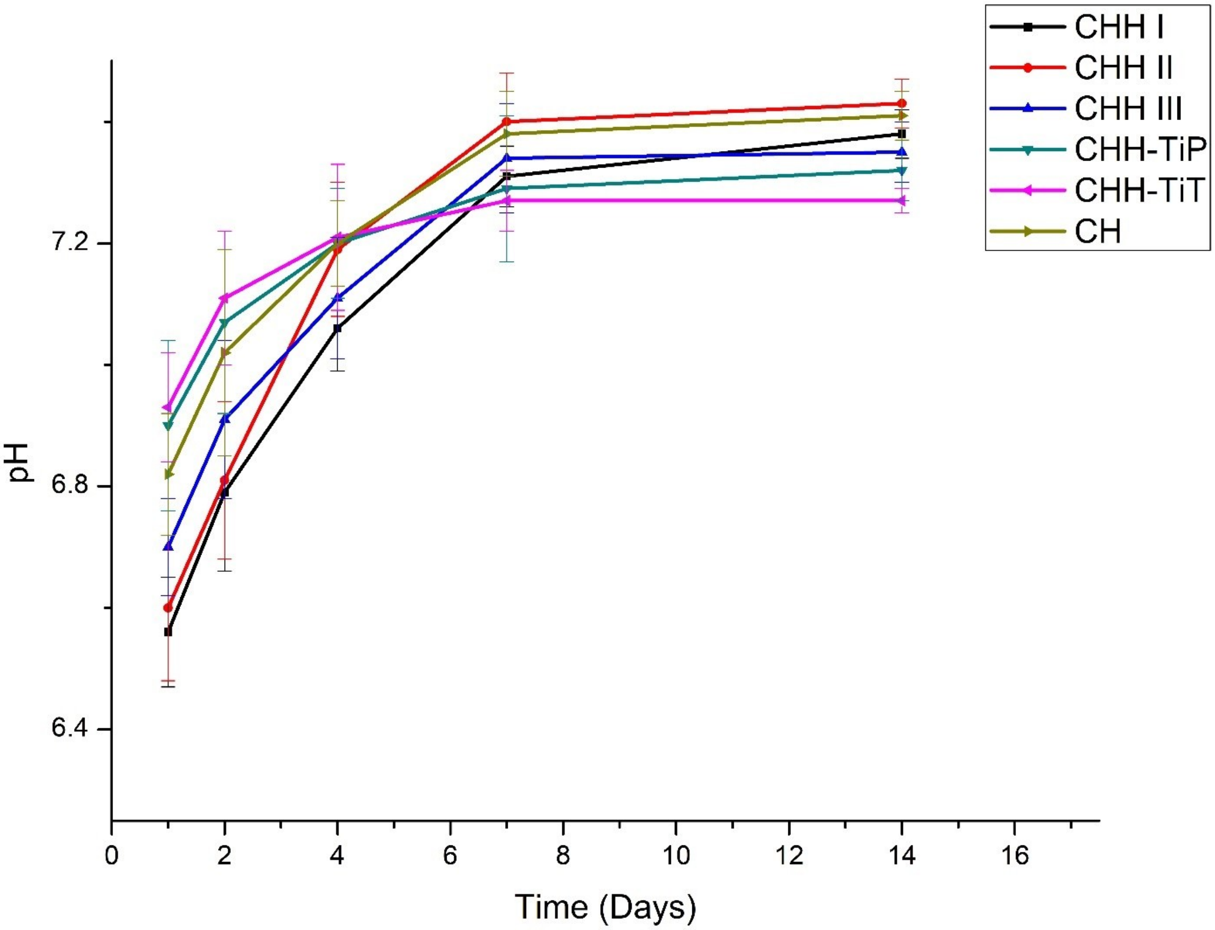

SI 3.12: pH studies of CHH I-III, CHH-TiP, CHH-TiT and CH. Data here is represented as mean of triplicate values ± S.D.

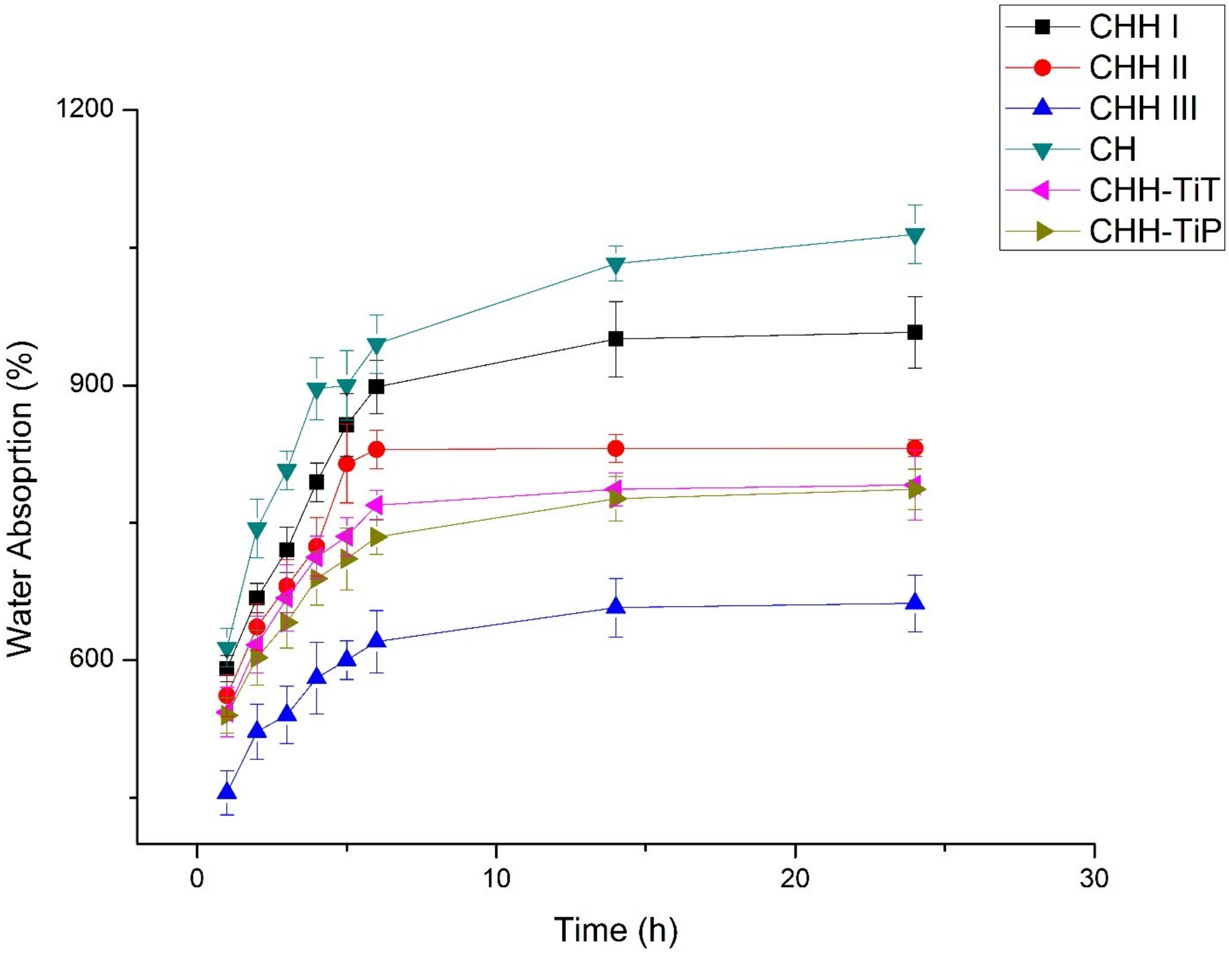

SI 3.13: Water absorption studies of CHH I-III, CHH-TiP, CHH-TiT and CH. Data here is represented as mean of triplicate values ± S.D.

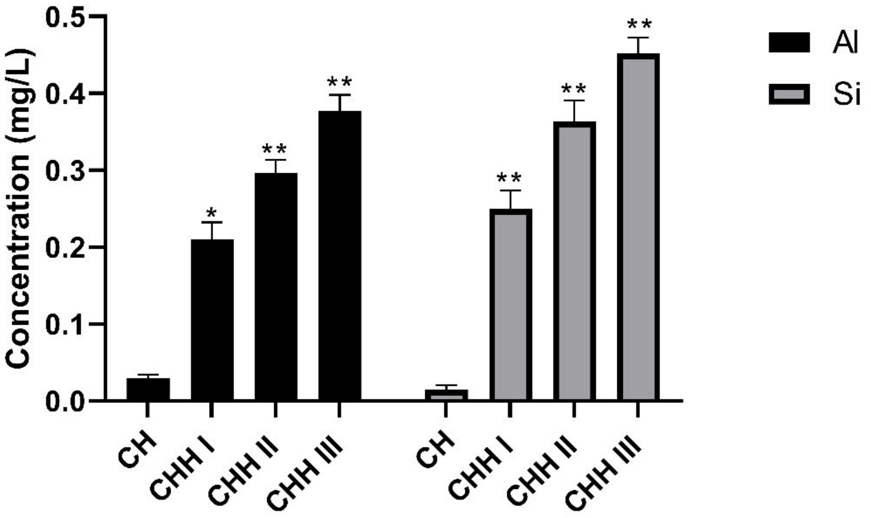

SI 3.14: Ion release study of CHH I-III and CH in PBS at Day 7 after initial immersion. Data is represented as means of triplicate wells ± SD, wherein * indicates $p < 0.05$ and ** indicates $p < 0.01$ when compared with CH film.
